# Prediction of hot spot residues at protein-protein interfaces by combining machine learning and energy-based methods

**DOI:** 10.1186/1471-2105-10-365

**Published:** 2009-10-30

**Authors:** Stefano Lise, Cedric Archambeau, Massimiliano Pontil, David T Jones

**Affiliations:** 1 Department of Computer Science, University College London, UK

## Abstract

**Background:**

Alanine scanning mutagenesis is a powerful experimental methodology for investigating the structural and energetic characteristics of protein complexes. Individual amino-acids are systematically mutated to alanine and changes in free energy of binding (ΔΔ*G*) measured. Several experiments have shown that protein-protein interactions are critically dependent on just a few residues ("hot spots") at the interface. Hot spots make a dominant contribution to the free energy of binding and if mutated they can disrupt the interaction. As mutagenesis studies require significant experimental efforts, there is a need for accurate and reliable computational methods. Such methods would also add to our understanding of the determinants of affinity and specificity in protein-protein recognition.

**Results:**

We present a novel computational strategy to identify hot spot residues, given the structure of a complex. We consider the basic energetic terms that contribute to hot spot interactions, i.e. van der Waals potentials, solvation energy, hydrogen bonds and Coulomb electrostatics. We treat them as input features and use machine learning algorithms such as Support Vector Machines and Gaussian Processes to optimally combine and integrate them, based on a set of training examples of alanine mutations. We show that our approach is effective in predicting hot spots and it compares favourably to other available methods. In particular we find the best performances using Transductive Support Vector Machines, a semi-supervised learning scheme. When hot spots are defined as those residues for which ΔΔ*G *≥ 2 kcal/mol, our method achieves a precision and a recall respectively of 56% and 65%.

**Conclusion:**

We have developed an hybrid scheme in which energy terms are used as input features of machine learning models. This strategy combines the strengths of machine learning and energy-based methods. Although so far these two types of approaches have mainly been applied separately to biomolecular problems, the results of our investigation indicate that there are substantial benefits to be gained by their integration.

## Background

Protein-protein interactions are central to most biological processes including for example cellular communication, gene regulation, and immune response [1]. The complexity of these processes, coupled with the intricate interaction networks that biomolecules form in a cell, requires proteins to be able to selectively bind to other proteins. Indeed, erroneous or disrupted protein interactions can be the causes of a number of diseases [2]. Elucidating the fundamental biophysical principles that govern molecular recognition and drive protein association is therefore a topic of primary importance in biomedical research. However, at present the energetic determinants of affinity and specificity in protein interfaces are still poorly understood and fundamental problems relating to the recognition process are yet to be solved. 

Knowledge of the three-dimensional (3D) structure of the complex provides much valuable information on the architecture and chemistry of a protein-protein interface, including the identity of residues in contact, the size and shape of the interface, the number of hydrogen bonds, and the presence of bound water molecules. On its own, however, the structure does not fully clarify the details of the energetics of binding, nor does it determine to what extent each residue modulates complex formation and contributes to the overall affinity and specificity. For example, understanding how particular amino-acid mutations affect binding would help explaining the causes of some diseases and possibly suggest a strategy to treat them [3,4]. For a more accurate description of protein-protein interaction and its effects, e.g., on a pathway or on a whole biological system, structural and thermodynamic analysis provide complementary information and both are necessary [1].

The thermodynamics of protein-protein interactions can be probed experimentally by alanine scanning mutagenesis [5]. Interface amino-acids are systematically replaced with alanine and the induced changes in binding free energy measured. As alanine amino acids do not have a side-chain beyond the *β*-carbon, this procedure in effect tests the importance of individual side-chain groups for complex formation, providing a map of the so-called functional epitope (to be distinguished from the structural epitope defined instead by all residues at the interface [6]). Results from a number of experiments indicate that only a small subset of contact residues contribute significantly to the binding free energy. These residues have been termed "hot spots" and if mutated they can disrupt the interaction. For the majority of interface residues instead, the effect of an alanine mutation is minimal (for a review on hot spots and their properties see, e.g., [7,8]).

In recent years, several computational approaches have been developed to predict hot spot residues in a protein complex structure (see, e.g., discussion in [4]). Accurate predictive models provide a valuable complement to experimental studies and add to our understanding of the factors that influence affinity and specificity in protein-protein interfaces. In addition, they potentially have important applications in the field of drug discovery. Protein-protein interfaces are in fact emerging as a prospective new class of therapeutic targets. Although dealing with protein binding epitopes is more challenging compared to, e.g., enzyme binding pockets, a number of studies have been successful in developing (drug-like) small molecules that bind at hot spots and inhibit complex formation. Reliable hot spots predictions can therefore represent the first step in rational drug design projects [3,4].

Most computational methods predict hot spots by simulating an alanine substitution and estimating the induced changes in binding free energy (ΔΔ*G*). One class of methods is based on molecular dynamics (MD) simulations [9,10], which makes them computationally rather expensive and difficult to apply on a large scale. A second class instead relies on empirically calibrated free energy functions [11,12], which include terms such as van der Waals and electrostatic interactions, hydrogen bonds and solvation energy. These terms are then combined linearly, with weights adjusted in order to best fit experimental mutagenesis data. As energies are evaluated on static structural configurations (as opposed to MD simulations, where free energies are ensemble averages), these latter methods are computationally much faster and reported results appear comparable to those from MD simulations [11]. More recently, machine learning approaches have also been applied to the problem of detecting hot spot residues [13,14].

In this paper, we propose a novel computational strategy to predict hot spot residues at protein-protein interfaces. Similarly to other energy-based methods, we consider the basic terms that contribute to hot spot interactions (van der Waals potentials, hydrogen bonding, electrostatic interactions and solvation energies). Rather than writing an explicit energy function from which we can then calculate ΔΔ*G*, we treat them as input features of a machine learning algorithm. The rationale beyond our approach is that the exact functional form for ΔΔ*G *is not known but it is reasonable to assume that it would incorporate these terms. Given a set of training examples of alanine mutation data, we use machine learning methods to deduce the functional properties of ΔΔ*G*.

We consider two conceptually different machine learning methods, Support Vector Machines (SVMs) [15] and Gaussian Processes (GPs) [16]. We compare our results to previous methods and in particular to the predictions of the Robetta server [11] for the same set of mutations. Robetta is a well established energy-based method which has become the *de facto *standard of comparison in the field. We show that our approach is significantly more accurate in identifying hot spots (here defined as those residues for which ΔΔ*G *≥ 2 kcal/mol). Among the tested methods, we find the best performances using Transductive Support Vector Machines (TSVMs), a semi-supervised learning version of SVMs. We investigate also the problem of estimating the actual value of ΔΔ*G *induced by an alanine mutation. This proves to be a rather challenging problem. Results from both SVM regression and GP models are comparable and in some aspects superior to those from the Robetta server. However they are also comparable to those obtained by simple models built by ordinary linear least squares fitting (LLSF). We point out shortcomings and limitations of our as well as other energy-based models.

## Results and Discussion

The problem we have investigated is the prediction of hot spot residues at a protein-protein interface, given the structure of the complex. Basic thermodynamic considerations show that in order to correctly estimate the binding free energy change ΔΔ*G *upon alanine substitution one should in principle consider its effects on the unbound state as well. Let *A *and *B *denote the unbound monomers and *AB *the complex. For convenience, we assume that the alanine mutation occurs on protein *A*. We further denote with (wt) the wild-type molecules and with (mut) the mutated molecules. We can then write

(1)

(2)

from which follows

(3)

Equation (3) highlights the dependence of ΔΔ*G *on both the bound and unbound states. In general, it is therefore not possible to explain binding free energy differences entirely in terms of changes or deletions of atomic contacts across the interface as mutations can destabilise the unbound state as well [4,7].

Previous computational methods evaluate the free energies for the wild type and mutated proteins on both bound and unbound structures and deduce ΔΔ*G *from these through eq (3). In principle four distinct free energy calculations are required which can be computationally demanding (and as a consequence approximations are often introduced). In our approach we aim instead to estimate directly ΔΔ*G *without calculating the free energy *G *in the four different states. We consider only the complex structure and make no attempt to model the unbound and/or mutated structures. The input variables to the machine learning algorithms are basic energy terms (van der Waals, hydrogen bond, electrostatic and desolvation potentials) calculated from the complex structure.

**Figure 1:**
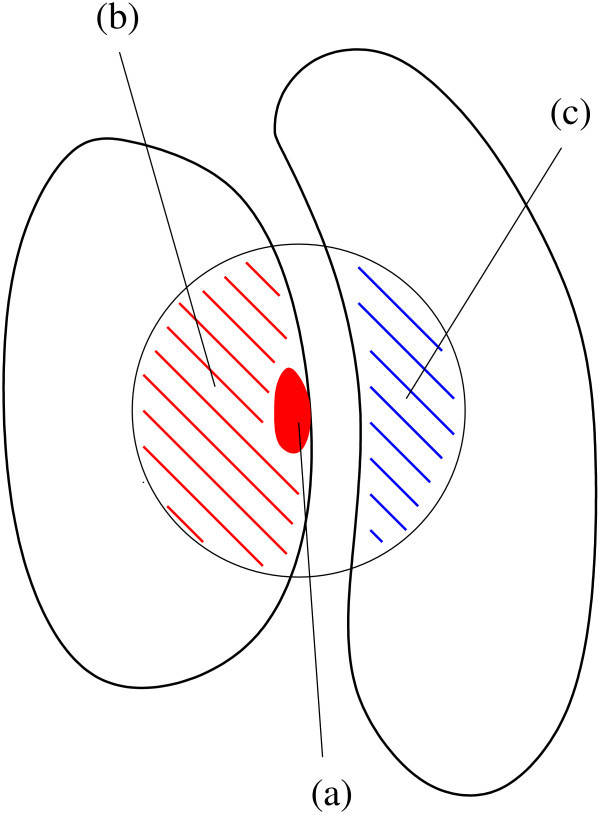
**Schematic overview of protein structural regions which define the different energy contributions**. The red filled area, (a), corresponds to side-chain atoms of the mutated residue; the red and blue striped regions, (b) and (c) respectively, correspond to atoms within 10 *Å *of the *C*_*β *_of the mutated residue. We distinguish 3 types of interactions: *side-chain inter-molecular *between (a) and (c), *environment inter-molecular *between (b) and (c), *side-chain intra-molecular *between (a) and (b).

We distinguish contributions from different structural regions in the protein complex (see Figure 1) and we associate to each of them 4 input features, corresponding to the basic energy terms mentioned above. It can be expected that side-chain atoms of the mutated residue contribute significantly to ΔΔ*G *as these are the atoms that are "cancelled" by an alanine mutation. In fact it can be shown (see discussion in Additional file 1) that if structural changes due to binding and to mutations are neglected, *side-chain inter-molecular energies *are the only contributions to ΔΔ*G*. The latter hypothesis however can not be expected to hold in general and therefore we have included other energy contributions as well.

The environment energy describes the (inter-molecular) interactions of those atoms that are located nearby the mutated residue. It aims to represent implicitly the plasticity of the local environment and its ability to rearrange. Local sequence and structure information are typically included as input features in a related problem, the prediction of stability changes upon mutations in monomeric proteins [17,18]. In the hot spot prediction problem, the inclusion of these terms describing the local environment possibly finds further justification in the O-ring hypothesis [19]. By analysing a large set of data it has been suggested that one important characteristic of hot spots is that they are surrounded by a set of residues (the O-ring) whose role is to shield them from the solvent. The environment energy might therefore capture this effect.

### Prediction of hot spot residues

We discuss first the binary classification problem, i.e. the problem of predicting if a residue is a hot spot (ΔΔ*G *≥ 2 kcal/mol) or not (ΔΔ*G *< 2 kcal/mol). Support Vector Machines (SVMs) are widely used tools in computational biology and well suited for the task (see [20,21] and references therein). As input features we consider the 8 terms associated with *side-chain inter-molecular *and *environment inter-molecular energies *(the addition of *side-chain intra-molecular energies *in this case does not improve the prediction accuracy). A summary of the results is reported in Table 1a according to various performance measures. The precision *P *is the fraction of true hot spots among the set of residues predicted to be hot spots; the recall *R *is the fraction of correctly identified hot spots relative to all those present in the data set; the *F*1 score is a weighted average of the precision and recall; the Matthews Correlation Coefficient (*MCC*) is a commonly used measure of the quality of binary classifications (see Methods section for more details). 

The results are significantly better than the ones expected for a random model. For example, the SVM classifier predicts a total of 96 hot spots of which 51 are true positives. Our data set consists of 349 alanine mutations of which 81 are hot spots. Choosing randomly 96 residues would therefore return 22 ± 4 true positives as can be estimated from a binomial distribution. The F1 score for a random predictor is *F*1_*ran*_≤ 0.37 and *F*1_*ran *_= 0.23 if the Recall is equal to the fraction of hot spots in the data set, i.e. *R*_*ran *_= 0.23 (see discussion in Methods section). For the SVM classifier we obtain *F*1 = 0.58 ± 0.02. Similarly, the Matthews correlation coefficient *MCC *= 0.44 ± 0.03 is significantly greater than zero, which is the random baseline. A simple chi-square test, *χ*^2 ^= *N *× *MCC*^2 ^= 67.6 (where *N *= 349 is the number of entries in the data set) [22], returns a highly significant result (chance probability < 10^-5^).

**Table 1:** Summary of results for the binary classification problem

	**Model**	**Precision**	**Recall**	**F1 score**	**MCC**
	
(a)	SVM	0.53 ± 0.03	0.63 ± 0.04	0.58 ± 0.02	0.44 ± 0.03
	TSVM	0.56 ± 0.03	0.65 ± 0.03	0.60 ± 0.02	0.47 ± 0.02
	GP	0.59	0.32	0.41	0.33
	Robetta_2_	0.52	0.47	0.49	0.35
	Robetta_1.8_	0.53	0.52	0.52	0.38
	Robetta_1_	0.39	0.75	0.52	0.34
	
(b)	SVM	0.64 ± 0.03	0.79 ± 0.05	0.71 ± 0.01	0.40 ± 0.03
	Robetta_1_	0.69	0.65	0.67	0.38

In (a) experimental hot spots are defined as those residues for which ΔΔ*G *≥ 2 kcal/mol, in (b) a threshold of 1 kcal/mol is used. SVM: Support Vector Machine, TSVM: Transductive SVM, GP: Gaussian Processes, : Robetta scores (estimated ΔΔ*G *values) which are greater than *x*_*th *_are considered as predicted hot spots. MCC is the Matthews correlation coefficient (see Methods section for definition of the various performance measures). Results for SVM, TSVM and GP have been obtained with a 16-fold cross-validation scheme. Results for Robetta have been retrieved from the server at .

Results quoted in Table 1a have been obtained through a 16-fold cross-validation strategy. We have verified that they are robust with respect to the cross-validation scheme employed, i.e. by using a more stringent 12-fold cross-validation the results do no change appreciably (see Additional file 2: Supplemental Table S1). As can be expected they tend to be slightly worse but the difference does not appear to be significant. A further control is to apply the models on their training sets and check if statistically the predictions are much better than on the test set. We have verified that the performances on the training sets are only marginally more accurate but still comparable to those on the test set (see Additional file 2: Supplemental Table S1). These considerations about the 12-fold cross-validation and the performances on the training sets suggest that there is no over-fitting of the data in our analysis and that the SVM model generalises to unseen data as quoted in Table 1a.

One concern related to the data set is that it contains a large number of immunoglobin domains and this might introduce a bias in the predictions. Ideally, to verify the extent of this problem, one should group all the immunoglobin domains in the same fold family when performing cross-validation and check if this affects significantly the performances. In practice this is not feasible, as the data set is relatively small and clustering the immunoglobins together would result in one fold dominating the database. However a first indication that there is no such bias towards predicting mutations on immunoglobins more accurately comes from the results of the 12-fold cross-validation. In this case, most (but not all) of the immunoglobin containing complexes are clustered together and the results do not change significantly. A second, *a posteriori *check is to analyse classification predictions separately for complexes containing an immunoglobin domain and those without. If there were a bias, we would expect a better performance on the former compared to the latter class but instead we obtain *P *= 0.55 ± 0.03, *R *= 0.60 ± 0.05, *F*1 = 0.57 ± 0.03, *MCC *= 0.40 ± 0.05 and *P *= 0.52 ± 0.03, *R *= 0.66 ± 0.02, *F*1 = 0.58 ± 0.02, *MCC *= 0.46 ± 0.03 respectively.

It is instructive to analyse in more detail what are the major contributions to the prediction accuracy of the SVM model. For example we have trained the SVM separately with the side-chain and the environment features, denoted respectively SVM-sc and SVM-env. The results are reported in Table 2. Although both models individually perform reasonably well, it is clear that their combination is superior. In particular, it seems that adding the environment terms to the side-chain terms improves the precision *P*, reducing the number of false positive (the recall *R *is substantially unchanged).

**Table 2:** Results for SVMs trained on a subset of features

**Method**	**Precision**	**Recall**	**F1 score**	**MCC**
SVM-sc	0.47 ± 0.02	0.64 ± 0.03	0.54 ± 0.02	0.38 ± 0.02
SVM-env	0.43 ± 0.03	0.72 ± 0.05	0.54 ± 0.03	0.37 ± 0.04

SVM-sc: only side chains terms included; SVM-env: only environment terms included.

As our SVM models are based on a linear kernel, the scoring function that discriminates hot spots from neutral residues is a simple linear combination of the energy terms. The associated weights can be computed based on the SVM models and are reported in Table 3. The weights roughly reflect the level of correlation that exists between the observed ΔΔ*G *values and each energy term (see Additional file 2: Supplemental Table S2). They provide a first indication about the relative importance of each of the 8 features. For example, the side-chain van der Waals term emerges as the most important one, whereas electrostatic terms appear to contribute only marginally.

**Table 3:** Weight of energy terms in the scoring function

**Feature (energy term)**	**Weight in SVM**	**Weight in TSVM**
Side-chain van der Waals	0.29 ± 0.05	0.37 ± 0.08
Side-chain hydrogen bond	0.15 ± 0.01	0.24 ± 0.03
Side-chain electrostatics	0.07 ± 0.03	0.08 ± 0.02
Side-chain desolvation	0.22 ± 0.01	0.19 ± 0.05
Environment van der Waals	0.17 ± 0.01	0.12 ± 0.04
Environment hydrogen bond	0.22 ± 0.04	0.24 ± 0.03
Environment electrostatics	0.07 ± 0.02	0.02 ± 0.03
Environment desolvation	0.12 ± 0.04	0.16 ± 0.04
Threshold	0.31 ± 0.07	0.90 ± 0.06

We report the absolute value of the weight associated to each feature in the scoring function as determined by SVM and TSVM, together with the threshold that defines the decision boundary.

To further validate these insights, we have trained the SVMs excluding one energy term at a time. Omission of the side-chain van der Waals potential leads to a consistent drop in accuracy, confirming the importance of this term. Similar outcomes are also observed when side-chain hydrogen bond, side-chain desolvation or environment desolvation potentials are excluded (see Table 4), suggesting a critical role of these 4 energy terms in determining hot spot residues. On the contrary, the two electrostatic terms, environment van der Waals and environment hydrogen bond potentials do not appear to be strictly necessary and their omission does not significantly affect the quality of the predictions (see Additional file 2: Supplemental Table S3).

**Table 4:** Results for SVMs trained excluding one feature

**Excluded feature**	**Precision**	**Recall**	**F1 score**	**MCC**
Side-chain van der Waals	0.49 ± 0.04	0.62 ± 0.03	0.54 ± 0.02	0.39 ± 0.02
Side-chain hydrogen bond	0.50 ± 0.04	0.58 ± 0.05	0.54 ± 0.02	0.39 ± 0.03
Side-chain desolvation	0.49 ± 0.03	0.63 ± 0.04	0.55 ± 0.02	0.40 ± 0.03
Environment desolvation	0.50 ± 0.05	0.59 ± 0.05	0.54 ± 0.04	0.39 ± 0.04

Here, only features that if omitted lead to a decrease in performance are reported (see Additional file 2: Supplemental Table S3 for all features).

The limited contributions from the electrostatic terms can be ascribed to their weak correlations to the target outputs, i.e. to the observed ΔΔ*G *values (see Additional file 2: Supplemental Table S2). The non essentiality of the environment van der Waals and hydrogen bond potentials seems instead to derive from the fairly high correlation existing between these two terms and the environment desolvation potentials (see Additional file 2: Supplemental Table S2). It suggests that if one of these two terms is missing the latter can effectively substitute for it (possibly with some readjustment from the other remaining terms as well). It is important to underline that these results do not imply that the mentioned 4 terms play no role and can be altogether ignored. Indeed omission of pairs of features can lead to a significant decrease in the performance.

The importance of the side-chain van der Waals term agrees with the observation that hot spot atoms form good packing interactions with the partner proteins [23-25]. The side-chain hydrogen bond term was found to provide a major contribution also in [11]. The role of the desolvation potential seems to support the O-ring hypothesis and the importance of shielding the interactions from the solvent. Exclusion of the solvent leads to a lower effective dielectric thereby increasing the strength of an interaction. In this respect it is somewhat surprising that electrostatics does not emerge as a key component in our model. Although a similar result was found in ref [11], it is possible that a better description of electrostatic effects is required, for example either by solving the Poisson-Boltzmann equation or through the generalised Born model. 

The above considerations indicate that although some terms are more important than others there is no single feature that makes a dominant contribution. Rather, it seems it is the balanced combination of terms in the SVM model that allows the detection of hot spots. This possibly provides a justification of why this prediction problem is hard. It also support the claim that there is no simple patterns of hydrophobicity, shape or charge that can be used to identify hot spots [7].

To further examine the reliability and usefulness of our approach, we have compared our predictions with the predictions of the Robetta server on the same set of mutations. The server returns an estimated value for ΔΔ*G *based on an all-atom free energy function. It can then be turned into a binary classifier by labelling a residue as a predicted hot spot if ΔΔ*G*_*calc *_≥ *x*_*th *_kcal/mol, where *x*_*th *_is some threshold value. In the following we denote with  such a classifier. Given our definition of hot spots, a natural choice is *x*_*th *_= 2. We have however also explored other values for *x*_*th *_because Robetta in its original implementation defines hot spots using a threshold of 1 kcal/mol and is therefore not optimised for our hot spot definition. We find indeed that more accurate predictions are obtained by setting *x*_*th *_= 1.8. Results are reported in Table 1a. By comparing them to those for our SVM approach, it can be deduced that the latter yields a substantial improvement. We emphasise though that the comparison is not entirely fair: the Robetta method has been designed to predict the actual value of ΔΔ*G *and as we will discuss below this a considerably more difficult problem than binary classification.

As mentioned above, in the original Robetta paper [11] a threshold of 1 kcal/mol was used to define experimental and predicted hot spots. With this definition, our data set is more balanced with respect to the number of positive and negative examples, respectively 165 (hot spots) and 184 (non hot spots). We have trained and tested our method with this hot spot definition as well and we report a summary of the results in Table 1b. Interestingly, in this case we obtain the best predictions by including the *side-chain intra-molecular energy *terms as well. Compared to the Robetta server (i.e. Robetta_1 _in our notation), our method appears to perform marginally better but the difference might not be statistically significant and it is certainly less pronounced than in the case of a 2 kcal/mol threshold (see Table 1).

Recently, a machine learning approach to predict hot spot residues has been presented [13]. It is based on decision trees and trained on features such as geometrical shape and biochemical properties (e.g. atomic contacts, hydrogen bonds and salt bridges). The predictive performance of the method has been estimated to be *P *= 0.49, *R *= 0.58 and *F*1 = 0.53 (*F*1 = 0.55 if the method is combined with the Robetta server). Comparing methods on the basis of quoted results is problematic as data sets and cross validation strategies differ. The data sets of alanine mutations used in [13] and in our investigation are not identical but they do overlap substantially (indeed very similar performance scores are obtained by applying Robetta_2 _to the two data sets, see Table 3 in [13] and Table 1a in this paper). The higher performance scores we obtain might therefore reflect a genuine improvement in hot spots prediction accuracy. The reason behind this improvement possibly lies in the inclusion of the environment energy terms in our method. Indeed results for SVM-sc in Table 2 appear comparable to those reported in [13]. Besides SVMs, we have also tested Gaussian Processes (GP) models for the classification task but found results with overall lower accuracy (see Table 1a).

After our manuscript was submitted and while still under review, two new studies on the same problem of hot spot prediction at protein-protein interfaces have been published [26,27]. It is not straightforward to assess how our method performs compared to them. We note for example that on data sets assembled from ASEdb the reported F1 score for the methods in [26] and [27] are respectively *F*1 = 0.65 and *F*1 = 0.57. However, on the same two data sets Robetta_2 _achieves respectively *F*1 = 0.55 and *F*1 = 0.59, which are both substantially higher than the value it obtains on our data set (*F*1 = 0.49). This suggests that our method and those in [26] and [27] can not be compared on the basis of the quoted results alone. 

We have experimented with introducing unlabelled data in the training set, a problem that is often refereed to as *semi-supervised learning*. Unlabelled data can sometimes improve a classifier by providing a more reliable decision boundary, for example by requiring that it lies (in the input features space) in region of low density. Transductive SVM have been developed to work in a semi-supervised learning setting and take advantage of the information content in unannotated data. In our case unlabelled data correspond to interface residues for which ΔΔ*G *is not known. As reported in Table 1a, we find TSVMs return marginally improved predictions although the difference might not be entirely significant. One possible explanation for this improved performance is that the inclusion of unlabelled data makes the training sets in the cross-validation procedia more balanced and statistically more uniform.

We have analysed the hot spot predictions by grouping mutations according to the amino acid type (see Table 5). We observe a good accuracy over all amino acid types, ranging between 0.64 and 0.94 (if we exclude Cys and Met residues for which not enough data are available). Predicted hot spots can e.g. be charged residues such Lys and Asp or hydrophobic, aromatic residues such as Tyr and Trp. This suggests that our model is not biased toward a single amino type or property (e.g. hydrophobic or charged residues) but rather it captures some composite properties characterising hot spots.

**Table 5:** Analysis of hot spot predictions for each amino acid type

**Mutated amino acid**	**N_*mut*_**	**TP**	**TN**	**FP**	**FN**	**P**	**R**	**F1**	**A**
Arg	33	5	16	10	2	0.33	0.71	0.45	0.64
Asn	22	3	15	1	3	0.75	0.50	0.60	0.82
Asp	29	7	15	5	2	0.58	0.78	0.67	0.76
Cys	1	0	1	0	0	NA	NA	NA	1.00
Gln	21	1	17	2	1	0.33	0.50	0.40	0.86
Glu	31	1	22	4	4	0.20	0.20	0.20	0.74
His	13	1	11	1	0	0.50	1.00	0.67	0.92
Ile	15	0	11	0	4	NA	0.00	NA	0.73
Leu	10	0	9	0	1	NA	0.00	0.00	0.90
Lys	32	10	20	1	1	0.91	0.91	0.91	0.94
Met	2	0	1	1	0	0.00	NA	NA	0.50
Phe	11	1	8	1	1	0.50	0.50	0.50	0.82
Ser	28	1	22	5	0	0.17	1.00	0.29	0.82
Thr	24	0	21	2	1	0.00	0.00	0.00	0.88
Trp	23	5	13	1	4	0.83	0.56	0.67	0.78
Tyr	44	17	17	7	3	0.71	0.85	0.77	0.77
Val	10	1	7	1	1	0.50	0.50	0.50	0.80
All	349	53	226	42	28	0.56	0.65	0.60	0.80

N_*mut*_: number of mutations, TP: true positives, TN: true negatives, FP: false positives, FN = false negatives; P: precision, R: recall, F1: F1 score, A: accuracy = .

**Figure 2:**
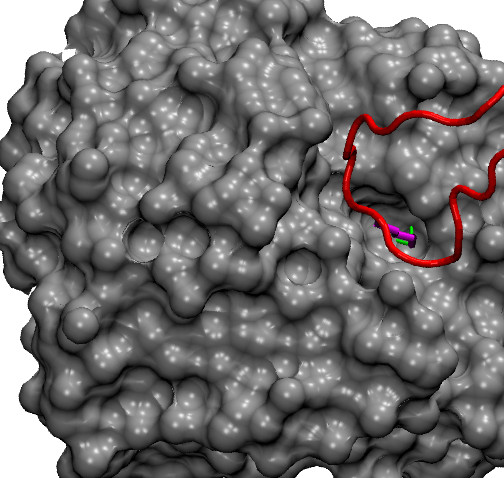
**Example of a lysine hot spot**. A detail of the interaction between trypsin (in grey, surface representation) and trypsin inhibitor (in red) is shown (pdb code 2PTC). The side chain of the hot spot Lys15 from the inhibitor (in magenta, ΔΔ*G *= 10 kcal/mol) fits into a hole on the trypsin surface. The *ζ*-nitrogen of the Lys side chain forms two hydrogen bonds (highlighted in green) with the residue Ser190 from trypsin.

The most accurate predictions are obtained for mutations involving Lys. This is a positively charged, polar amino acid capable of forming up to 3 hydrogen bonds with the amino group at the end of its side-chain; its side chain contains 4 carbon atoms which confer to it a partial hydrophobic character. Lys is among the more frequent amino acids in hot spots (see Table 6). As an "extreme" example of a mutation involving Lys we show in Figure 2 the trypsin-trypsin inhibitor complex. The residue Lys15 from the inhibitor is a hot spot having the largest measured ΔΔ*G *in our data set (ΔΔ*G *= 10 kcal/mol). Our correct positive prediction is driven mainly by the side-chain van der Waals and hydrogen bond energies. Arginine too is a positively charged, polar amino acid and its side-chain can form up to 5 hydrogen bonds. Predictions however are not as accurate as for Lys with a fair number of false positives. By inspecting these false predictions individually, it emerges that often the positive scores are driven by a large favourable side chain hydrogen bond energy. It is possible that in fact this term is over-estimated as the loss of some of the hydrogen bonds upon alanine mutation might be (at least partially) mitigated by, e.g., the inclusion of water molecules at the interface.

**Table 6:** Distribution of amino acid types in data set

**Mutated amino acid**	**In data set**		**Hot spots (ΔΔ*G *≥ 2 kcal/mol)**			**Enrichment in hot spots**
			
	**(Number)**	**(%)**	**(Number)**	**(%)**^(*a*)^	**(%)**^(*b*)^	
Arg	33	9.46	7	21.21	8.64	0.91
Asn	22	6.30	6	27.27	7.41	1.18
Asp	29	8.31	9	31.03	11.11	1.34
Cys	1	0.29	0	0.00	0.00	0.00
Gln	21	6.02	2	9.52	2.47	0.41
Glu	31	8.88	5	16.13	6.17	0.69
His	13	3.72	1	7.69	1.23	0.33
Ile	15	4.30	4	26.67	4.94	1.15
Leu	10	2.87	1	10.00	1.23	0.43
Lys	32	9.17	11	34.38	13.58	1.48
Met	2	0.57	0	0.00	0.00	0.00
Phe	11	3.15	2	18.18	2.47	0.78
Ser	28	8.02	1	3.57	1.23	0.15
Thr	24	6.88	1	4.17	1.23	0.18
Trp	23	6.59	9	39.13	11.11	1.69
Tyr	44	12.61	20	45.45	24.69	1.96
Val	10	2.87	2	20.00	2.47	0.86

The number and percentage of amino acids in our data set are shown. The number and percentage of hot spots for each amino acid type is also reported. For a given amino acid type, (%)^(*a*) ^is the percentage of hot spots with respect to the residues of that type in the data set (i.e. entry in column 4 divided by the corresponding entry in column 2); (%)^(*b*) ^is the percentage of hot spots of that type with respect to all hot spots in the data set (i.e. entry in column 4 divided by the sum of all entries in column 4). Enrichment in hot spots is calculated as the ratio of the frequency of a given residue type in hot spots (column 6) over the frequency of the same amino acid type in the whole data set (column 3). This table should be compared with Table 2 in [19]. Note that proline and glycine are not included in our data set.

**Figure 3:**
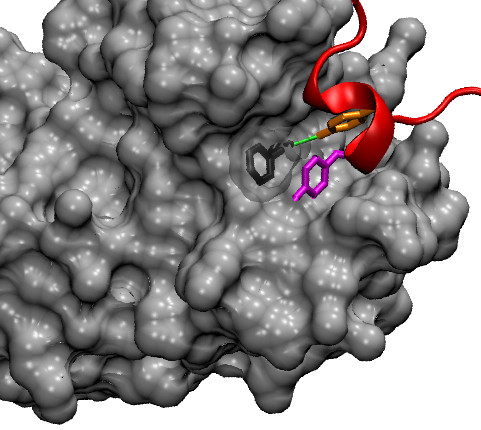
**Examples of tyrosine hot spots**. A detail of the interaction between colicin E9 immunity protein (in red) and colicin E9 DNase (in grey, surface representation) is shown (pdb code 1BXI). The side chain of the hot spots Tyr54 (ΔΔ*G *= 4.83 kcal/mol) and Tyr55 (ΔΔ*G *= 4.63 kcal/mol) are highlighted by a stick representation and are coloured in orange and magenta respectively. They fit into a groove of the E9 DNase surface. The residue Phe86 of E9 DNase is also displayed by sticks (dark grey with transparent surface representation). The hydroxyl group of Tyr54 is hydrogen bonded with the main-chain oxygen of Phe86 (green line); the aromatic ring of Tyr55 forms a stacking interaction with the aromatic ring of Phe86.

Tryptophan and tyrosine are also common in hot spots [19] (see Table 6). They are both aromatic, hydrophobic amino acid (Trp is more hydrophobic than Tyr as the latter contains a hydroxyl group) and are both capable of forming one hydrogen bond (see Figure 3 for two examples of Tyr hot spots). In general, our model appear to predict fairly accurately Trp and Tyr hot spots (see Table 5). In this respect it is worth noting that 3 false positive and 3 false negative predictions respectively for Tyr and Trp are found in the same complex, formed by the Interferon-*γ *Receptor and Antibody A6 (pdb code 1JRH). As discussed in [28] this is a difficult case, with the individual monomers likely to undergo significant conformational changes upon binding. Indeed predictions for this complex are among the less accurate we obtain (see Additional file 2: Supplemental Table S4). It has previously been noted that tyrosine is much more likely to be found in hot spots than phenylalanine, despite the two residues being similar and with nearly identical volumes. Presumably, this is due to the ability of tyrosine (and not phenylalanine) to hydrogen bond. It is reassuring that our model is able to reproduce this empirical observation fairly well (note that the identity of the mutated amino acid is not an explicit input feature).

For some amino acid types, our model has some clear limitations. For example, it performs poorly on mutations involving Glu despite it does reasonably well on Asp mutations. It has been observed that Asp is found more frequently in hot spots than Glu and this might be related to differences in side-chain conformational entropy [19]. This possibly also explains the low accuracy of our predictions, given that our method does not consider any entropic term. Another difficult case is isoleucine. Ile is an aliphatic, hydrophobic amino acid and similarly to Leu our method predicts no hot spots for this residue. In fact whereas Leu is rarely found in hot spots, Ile is actually enriched. Our model fails to distinguish between these two residues. In theory, one could think of exploiting the amino acid identity as input feature too and of building a model for each different amino acid. In practice, at present this is not feasible as there are not enough mutational data available.

### Prediction of ΔΔ*G *values

**Figure 4:**
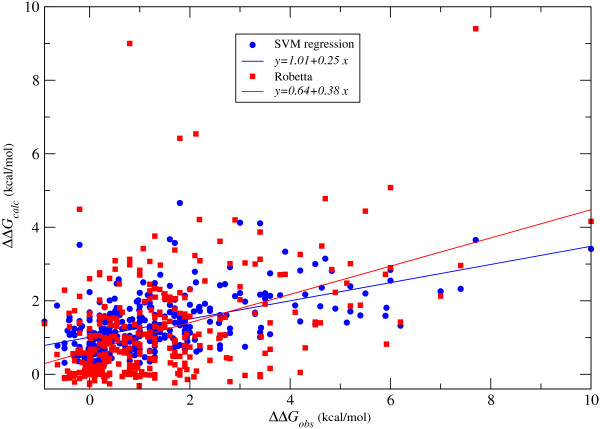
**Predicted versus observed changes in binding free energy ΔΔ*G***. In blue are the predictions from SVM regression, in red those from Robetta. The continuous lines correspond to linear fits between ΔΔ*G*_*calc *_and ΔΔ*G*_*obs*_, colour coded as the predictions (the respective equations are reported in the legend box).

We turn now to the more complicated regression problem, i.e. the prediction of the actual value of ΔΔ*G *induced by an alanine substitution. We have found beneficial in this case to include the *intra-molecular *energy terms as well. The results are summarised in Table 7. Both SVM regression and GP models compare favourably to the the Robetta server, although the difference is probably only marginal. Figure 4 shows the scatter plot of the predicted versus the observed ΔΔ*G*, both for SVM regression and for the Robetta server. SVM regression returns a lower root mean square error and an higher correlation coefficient. On the other hand, the regression line in Figure 4 is closer to the ideal case (i.e. slope and intercept equal to one and zero respectively) for the Robetta predictions. Notice that for some choices of hyper-parameters SVMs do return solutions with slope and intercept of the regression line closer to the Robetta ones. For these solutions the root mean square error and correlation coefficient are also similar to those derived from Robetta.

**Table 7:** Summary of results for the regression problem

**Method**	***RMSE***	***r***	**Precision**	**Recall**	**F1 score**	***MCC***
SVM	1.34 ± 0.05	0.54 ± 0.03	0.62 ± 0.05	0.44 ± 0.05	0.52 ± 0.03	0.41 ± 0.04
GP	1.36	0.52	0.58	0.47	0.52	0.39
Robetta	1.52	0.47	0.52	0.47	0.49	0.35
LLSF	1.36	0.51	0.54	0.46	0.50	0.36
LLSF^(*a*)^	1.45	0.44	0.54	0.42	0.47	0.34
LLSF^(*b*)^	1.43	0.47	0.55	0.43	0.48	0.35

SVM: Support Vector Machine, GP: Gaussian Processes, LLSF: Linear Least Squares Fit, LLSF^(*a*)^: Linear Least Squares Fit with inter-molecular side-chain van der Waals as only input, LLSF^(*b*)^: Linear Least Squares Fit with inter-molecular side-chain van der Waals and hydrogen bond as inputs. RMSE stands for root mean squared error, *r *for correlation coefficient and MCC for Matthews correlation coefficient (see Methods section for definition of the performance measures).

All tested methods appear limited in the accuracy they can achieve. For example, if < ΔΔ*G *> is the average outcome of an alanine mutation simply by predicting ΔΔ*G *= < ΔΔ*G *> independently of the input features, one would obtain a *RMSE *= 1.58 kcal/mol. Of course in this case the correlation coefficient would be zero but this simple consideration suggests that root mean square errors of the order of those both we and other methods obtain are not extraordinary. In fact our results are comparable to those from a model built with an ordinary linear least squares fit (LLSF) as reported in Table 7. Compared to LLSF, SVM regression might still be slightly more accurate possibly because it is less sensitive to the effect of outlying points.

We have investigated also "minimal" version of the LLSF models, based on only one or two energy terms as inputs. This analysis highlights which are the major contributions to the models and reveals that, similarly to the binary classification problem, the most important term is the packing of the mutated side-chain against the partner protein. A model based on just the side-chain inter-molecular van der Waals energy achieves results that are worse but still comparable to the full model (see Table 7). Adding the hydrogen bond term the accuracy improves only marginally. We suggest therefore that the simple side-chain van der Waals model could be used in future as a benchmark to gauge advancements in this regression problem. 

Progress in this field will probably require the development of novel approaches. Based also on our results, it seems likely that it will involve modelling of both the mutated and unbound structures. This indeed might be the major limiting factor in our strategy. Some improvements might also be expected by a more accurate description of the different energy terms. For example a more rigorous treatment of solvent effects on electrostatics might be necessary. Additionally, terms can be introduced to explicitly account for the structural plasticity and adaptability of hot spot regions, e.g. by means of normal mode analysis or dynamical simulations. It is also possible that the addition of evolutionary information can lead to more reliable predictions. Indeed, in a recent investigation it has been highlighted that hot spots tend to establish conserved physico-chemical interactions across homologous interfaces [29]. However, in order to achieve major improvements it will be essential to rely on a larger data set of alanine mutations.

## Conclusion

In this study we have presented a novel computational approach to identify hot spot residues in protein-protein interfaces, given the structure of the complex. Basic energy terms are used as input features of machine learning algorithms such as SVMs. We have shown that we can identify hot spot residues with reasonable accuracy, substantially improving over, for example, the Robetta server [11]. The prediction of the actual value of ΔΔ*G *is instead still problematic. At present there seems to be no computational method that is able to predict the consequences of an alanine mutation to within chemical accuracy (i.e. with an error of the order of 1 kcal/mol or lower) and that at the same time is quick enough to be applied on a large scale.

We have developed an hybrid scheme that attempts to combines the strengths of machine learning and energy-based methods. Although so far these two approaches have mainly been applied separately to biomolecular problems, the results of our investigation indicate that there can be substantial benefits to be gained by their integration. Interestingly, a recent paper has applied somewhat similar concepts to the prediction of stability changes upon mutation in monomeric proteins [30], by combining attributes derived from a knowledge-based potential (rather than physical potentials as in our case) with machine learning algorithms.

The prediction of hot spot residues is a difficult but important problem. It represents a test of our understanding of the physical basis of affinity and specificity in protein-protein interactions. It is conceivable that progress on these aspects will lead to advancements in the docking problem as well, i.e. the prediction of the structure of a complex given the structures of the constituent proteins. For structural prediction purposes however the goal is to be able to locate hot spot residues in unbound proteins. Encouragingly, two recent studies have reported some success in predicting hot spots without prior structural knowledge of the complex [28,31]. It would be interesting to verify if an approach conceptually similar to the one presented here could be applied to detect hot spots in unbound proteins as well. There is evidence that binding residues are often located in energetic unusual environments and contribute unfavourably to protein stability [32-34], suggesting that energy features might indeed prove effective. 

The methodological approach we have outlined here is in principle rather general and could be applicable to other problems as well. For example it should be almost immediate to test it on monomeric proteins. The effects of alanine mutations in this case could be evaluated both in terms of stability changes and of modifications to the folding pathway (Φ-values). More general mutations could also be assessed although in this case it might be necessary to model the new amino acid. With regard to the docking problem mentioned above, in recent years many efforts have been directed towards the development of computational methods that can discriminate near-native structures among decoy sets. A classifier trained on energy features might be apt to the task. Another interesting and extremely challenging problem is the prediction of the thermodynamic and kinetic parameters which characterise the interaction between a ligand and a protein [35]. Combining physical potentials and machine learning algorithms might provide important insights which can then have implications, e.g., for the rational design of drug compounds.

## Methods

### Data Sets

The data set consists of protein complexes whose structures have been solved by *X*-ray crystallography and for which alanine mutational data are available. Structures are obtained from the Protein Data Bank (PDB) [36]. Alanine mutation data are collected from the Alanine Scanning Energetics database (ASEdb) [37] and from previous publications [11,13]. We have only considered protein-protein interactions involving an extended interface. Mutation data related to protein-peptide complexes have not been included. For each reported mutation we have verified the original reference and checked that the residue mapping from sequence to structure is consistent (few discrepancies have been found). Only mutations occurring at the complex interface have been retained. Interface residues are defined as those having at least one heavy atom within 5 *Å *of an heavy atom in the binding partner. Similarly, two residues across the interface are considered in contact if any of their heavy atoms are within 5 *Å*.

To ensure that the data set is sufficiently diverse and representative of protein-protein interfaces in general, we have analysed the complexes in terms of interacting domains and of location of the binding interface (i.e. which residues and residue contacts are at the interface). Domain structures can be classified according to CATH [38], which follows a hierarchical scheme. The first five levels in this hierarchy are Class, Architecture, Topology (fold), Homologous superfamily and Sequence family (clustered at a threshold of 35% identity). We have required that no two pairs of interacting domains have the same CATH numbers at the S-level. If two domain pairs have the same CATH numbers, we verify if they use the same or a different interface to interact. In the latter case, both structure are included in the data set (in practise this issue arises for only one pair of structures, PDB codes 3HFM and 1VFB).

The data set contains 20 protein complex structures. Following previous publications [19], we define hot spots as those alanine mutations for which ΔΔ*G *≥ 2 kcal/mol (ΔΔ*G *is the change in binding free energy). In total the data set comprises 349 mutations, of which 81 correspond to hot spots. Some studies such as for example in the Robetta paper [11] define hot spots using instead a threshold of 1 kcal/mol. According to this definition, in our data set there would be 165 hot spots and 184 non hot spots. We have tested our approach in this case as well and report the results in Table 1b.

The list of the 20 protein structures and their interacting domains is reported in Additional file 2: Supplemental Tables S5 and S6. Note that homologous protein complexes are present in our data set but at least one of the proteins involved has less than 35% sequence similarity to its homologue. We remark that in these cases only a limited number of mutations occurs at equivalent sequence positions, as deduced from the alignment, and even removing them from the data set does not affect the overall results. As can be noted, the data set is dominated by the immunoglobin superfamily (10 structures out of 20 comprise at least one immunoglobin domain). We have verified *a posteriori *that this does not introduce a bias and that mutations on immunoglobins are not predicted with higher accuracy than on other proteins (see Results and Discussion section).

A detailed list of the individual mutations with their respective ΔΔ*G *is reported in Additional file 3: Data set S1. We have analysed the distribution of amino acid types that occur in hot spots, in relation to the distribution of amino acid types in the database (Table 6). A similar analysis was performed in ref [19] and the results roughly agree with ours (e.g. tryptophan and tyrosine are among the most frequent amino acids in hot spots). We find however some notable differences, e.g. hot spots are not enriched in arginine (they are instead substantially enriched in lysine). Whereas the analysis in ref [19] was based on the whole ASEdb database, we have filtered out redundant entries and included only mutations located at a protein-protein interface for which the crystal structure of the complex is available. This might be the origin of the observed differences.

In recent publications [13,26,27], mutation data extracted from the Binding Interface Database (BID) [39] have been used to test proposed hot spot prediction models. In our opinion, these data have several problems (e.g. they are not associated to ΔΔ*G *values) and it is questionable whether they are useful in assessing the predictive power of a method. Nonetheless, for completeness we have extracted from BID our own data set and tested our method on it. We report the results in Additional file 4 together with a discussion of our concerns about this data set.

#### Data clustering for cross-validation

Mutations in the same or homologous binding interfaces can not in general be expected to be unrelated. This constitutes a problem when estimating the performance of a model through, e.g., cross-validation as there might be some 'trivial' similarities between data in the training and the test sets. In accordance, to avoid any potential bias, we have grouped together mutations belonging to the same or homologous complexes and assigned them to the same cross-validation fold. For our purposes, we have considered as homologous two complexes that share at least one pair of interacting domains similarly classified at the H-level in CATH, i.e. domain pairs with the same first 4 CATH numbers.

The above protocol produces 16 clusters (see Additional file 2: Supplemental Table S6) and accordingly a 16-fold cross-validation strategy is employed to assess the performance of the method (see below for more details on cross-validation). In order to assess the robustness of the approach and the presence of a residual redundancy in the 16-fold partition of the data set, we have also tested more stringent clustering criteria, i.e. complexes are grouped together if at least one of the two following conditions is met

• Interacting domains pairs are the same at the T-level (i.e. have the same fold),

• Individual domains are the same at the S-level and use the same binding interface, irrespective of their interacting partners.

This latter set of criteria results in 12 clusters (see Additional file 2: Supplemental Table S6) and in a 12-fold cross-validation strategy.

### Input features: energy components

As input features for the machine learning algorithms we have used basic energy terms that have been found to be important for the stability of protein complexes. These are van der Waals potential, hydrogen bonds, Coulomb electrostatics and desolvation energy. For each of the four energy components we have separately calculated 3 different energy contributions (schematised in Figure 1):

• *Side-chain inter-molecular energies*: interaction energies between side-chain atoms of the mutated residue and atoms in the partner protein (respectively atoms in the red filled area and blue striped area in Figure 1).

• *Environment inter-molecular energies*: interaction energies between atoms in the two proteins that are within 10 *Å *of the *C*_*β *_of the mutated residue (respectively atoms in the red striped area and blue striped area in Figure 1). We do not include the contribution from the mutated side-chain in this term.

• *Side-chain intra-molecular energies*: interaction energies between side-chain atoms of the mutated residue and other atoms in the same protein (respectively atoms in the red filled area and red striped area in Figure 1).

In total therefore there are 12 input features (4 × 3), although e.g. for Support Vector Machines we have used only a subset of them as this would give better or equivalent performances to the full set (see below for more details).

The energy components are calculated from the PDB structures. Only heavy atoms are considered and hydrogen atoms, if present, have been discarded. The computation of the different energy terms draws on established force-fields [11,40,41] but it incorporates some adjustments. It is important to remark that all terms are pairwise additive and therefore their calculation is formally equivalent. Before applying machine learning methods input features are rescaled so that they vary within similar numerical ranges. In the classification problem, each feature is standardised by subtracting its mean and dividing by its standard deviation. For regression purposes instead features are rescaled by their respective quadratic means. The latter normalisation makes it easier to search for solutions with no constant term, i.e. such that ΔΔ*G *= 0 if all energy terms are zero.

#### van der Waals energy

The energy of van der Waals interactions between two atoms *i *and *j *is calculated using a "smoothed" 6 - 12 Lennard-Jones potential

(4)

where *r *is the distance between the two atoms and the parameters *r*_*ij *_and *ϵ*_*ij *_are taken from CHARMM19 force fields [40]. The parameter *r*_0 _= 0.5 *Å *has the effect of widening the region of maximum affinity and to reduce the potential energy at *r *= 0 to a finite value. It makes the potential less sensitive to the precise position of atoms and to minor coordinate errors and local clashes. A similar option is available in the AutoDock suite [42]. We have also introduced a long distance cut-off, i.e. *V*_*vdW*_(*r*) = 0 if *r *> 8 *Å*.

#### Electrostatics energy

Electrostatic interactions are evaluated with a screened Coulomb potential, in which the dielectric constant increases linearly with distance. The atomic partial charges have been taken from the CHARMM19 parameter set [40]. In the case of side-chain inter-molecular interactions the charged forms of Arg, Lys, Asp and Glu is used [40]. For the environment inter-molecular and side-chain intra-molecular interactions instead a neutralised form of the ionic groups in these residues is employed [41]. This choice reflects the consideration that salt bridges in deleted side-chains might have a significant impact on binding free energy changes. In all cases we consider a neutral form of His (calculated as an average over the two possible single protonated configurations). As we do not explicitly include hydrogen atoms, their partial charges are simply added to those of the heavy atom to which they are covalently bound (e.g. the partial charge on a backbone N atom is taken to be -0.1 = -0.35 + 0.25, where -0.35 and 0.25 are respectively the partial charges associated with a bare backbone N atom and its attached hydrogen).

#### Hydrogen bond energy

We have followed the approach outlined in [11]. The energy of a hydrogen bond is computed as a linear combination of a distance-dependent part and two angular dependent components. The distance dependent term is modelled with a 10-12 potential, whereas the angular dependencies are derived from the probability distributions observed in high-resolution crystal structures. The same energy function is used for side-chain/side-chain and side-chain/backbone hydrogen bonds but they are weighted differently. Side-chain/side-chain hydrogen bonds are further divided into three classes, depending on the extent of burial of participating residues. Weights for inter-molecular and intra-molecular hydrogen bonds are also different. Details and parametrisation can be found in [11].

In our implementation, we have introduced a smoothing parameter, *r*_0 _= 0.25 *Å*, in the distance dependent component, similarly to the van der Waals potential described above. We have not distinguished between *sp*^2 ^and *sp*^3 ^hybridised acceptor atoms, rather we have taken an average of the knowledge-based angular potential in these two cases. In ref [11] backbone/backbone hydrogen bonds are not included in the free energy function as alanine scanning does not directly probe them. There is therefore no reported weight associated to them. In our formulation instead backbone/backbone hydrogen bonds contribute to the environment inter-molecular energies. For simplicity we have associated them the same weight as for the side-chain/backbone hydrogen bonds. We have used the program HBPLUS [43] to determine the characteristics (i.e. atomic distances and angles) of hydrogen bonds in the complexes.

#### Desolvation energy

The desolvation energy is evaluated using an implicit solvation model [41], which decomposes the solvation free energy into a sum of pairwise atomic interactions. The calculation of the input features associated with desolvation energy is therefore formally equivalent to the other energy terms. We have used a set of improved solvation parameters described in [44].

### Support Vector Machine models

Support Vector Machines (SVMs) are playing an increasingly important role in the field of computational biology [20,21]. They can be applied both to classification and regression problems. In the context of our investigation the former corresponds to predict whether a residue is a hot spot or not, the latter to estimate the actual value of ΔΔ*G*. We have used the program package SVM^light ^[45], which is available at the website . The program provides several standard kernel functions and we have experimented with linear, polynomial and Gaussian kernels, and with different combinations of input features. For classification purposes, we find the best results using a linear kernel and a set of 8 input features corresponding to the side-chain and environment inter-molecular energies. Using either a polynomial or a Gaussian kernel and a different (possibly larger) combination of input features, the performance would not improve, so we have opted for the simpler model. In the regression task, the best results are obtained with a linear kernel and by adding the van der Waals term from the side-chain intra-molecular energies to the features set (9 input features in total). The bias parameter in the kernel has been set to zero.

Standard SVM classifiers follow a supervised ("inductive") learning approach, whereby a predictive model is built on the basis of available positive and negative examples. The SVM^light ^package implements also a semi-supervised learning algorithm, the so-called Transductive SVMs (TSVMs), in which unlabelled data are exploited in order to develop a better discriminatory model. In our case, unlabelled data correspond to interface residues that have not been mutated to alanine and for which therefore ΔΔ*G *is not known. The unlabelled data are extracted from the 20 protein complexes in the data set and used to integrate the original mutational data (maintaining the 16-fold partition described above for cross-validation). Optimal performance for TSVM classifier is obtained with a linear kernel and the same 8 features as for the SVM classifier.

#### Cross-Validation and Model Selection

SVMs have a number of tunable parameters (hyper-parameters) which should be chosen in order to achieve good generalisation performance and avoid over-fitting. For the classification task using a linear kernel, for example, two parameters, *C *and *j*, have to be set. The parameter *C *controls the trade off between the training error and the square norm of the weights associated to the features; the cost factor *j *determines how training errors on positive examples outweight errors on negative examples. An additional parameter is present in TSVMs, the fraction *p *of unlabelled examples to be classified into the positive class. In a regression setting the hyper-parameter *ϵ *controls the tolerance to errors. The optimal values for the hyper-parameters are not known in advance. In order to both evaluate fairly the performance of the methods and do not introduce biases in the choices of hyper-parameters, we have implemented a nested-loop cross-validation scheme [46] (see scheme in Additional file 2: Supplemental Figure S1) coupled with a grid search. The procedure ensures that hyper-parameters are selected without ever considering the performance on the test sets.

In nested-loop cross-validation, the data set is split into *n *folds (in our case *n *= 16 or *n *= 12). (*n *- 1) folds are used as the training set and the remaining fold is used as test set. The hyper-parameters are optimised on the training set, by applying a grid search and an internal (*n *- 1)-fold cross-validation strategy. The hyper-parameters that give the best cross-validated performance are selected and the associated model is then applied to the held-out test set. This process is repeated *n *times so that each of the original *n *folds is used as the test set once and predictions are obtained for each entry in data set. The procedure therefore consists of two nested cross-validation loops: an outer one for testing, an inner one for choosing hyper-parameters. In the inner cycle, we have assessed the model performance by means of the F1 score and the root-mean-square error (RMSE) for classification and regression tasks respectively (see below for more details).

For a given performance measure (e.g. the F1 score), we estimate its value *f *by considering the whole data set and by comparing predictions (obtained as described above) to the observed ΔΔ*G*. The associated statistical error is instead evaluated as follows. Let {*f*_*i*_}_*i *= 1... *n *_be the set of optimised values obtained on the n training sets, as a result of the inner loops of cross-validation. We then write

(5)

Note that the more standard procedure of calculating the score and its error from the average and standard deviation on the *n *test sets is not applicable in our case as individual test sets are rather small and of different sizes.

We have verified that our approach does not produce over-fitting of the data by comparing results obtained from a 16-fold and from a more stringent 12-fold cross-validation. A significant better performance for the 16-fold cross-validation would suggest over-fitting. In addition we have tested the *n *models on their own training sets. Better results can clearly be expected in this case with respect to the test set but a large difference would possibly indicate over-fitting of the data.

### Gaussian Process models

Gaussian Processes (GPs) are machine learning methods rooted in a Bayesian probabilistic framework [16]. They can be used for both regression and classification tasks. GPs assume that the data are a finite number of noisy observations generated by an unknown function. In line with Bayesian approaches [47], GPs assign a prior probability to each possible function which is then updated into a posterior probability in light of the observed data using Bayes' rule. Predictions on new data are made by a weighted average over all possible functions (models), the weights being equal to the posterior probabilities. Notice the different learning strategy between SVMs and GPs: SVMs determine one optimal model and make predictions based on it, GPs estimate a probability distribution over all possible models and predictions are model averages. One advantage of the latter approach is that it provides also confidence measures (i.e. error bars) on the predictions.

A GP is defined by a covariance function, which is the analogous of the kernel function in SVMs. Learning in GPs involves selecting a suitable covariance function for the problem at hand. As for SVMs, standard covariance functions include linear, polynomial and Gaussian functions. For both regression and classification we find the best results using a linear kernel and the full set of 12 input features. In the regression problem, the bias parameter of the kernel is set to zero. The data likelihood is assumed to be isotropic Gaussian. Notice that by using a linear kernel, a GP regression becomes equivalent to a Bayesian linear model with a Gaussian prior probability distribution over the linear coefficients. The GP classifier is computed based on the Laplace approximation [16] and using a logistic likelihood function. Intuitively, GP classification can be viewed as a generalisation of logistic regression (see e.g. [48]). For the calculations, we have used a software developed by one of us (CA).

#### Cross-Validation and Model Selection

Unlike SVMs, GPs do not require cross-validation to select the kernel hyper-parameters. They are learnt from the data by maximising a quantity called the log-marginal likelihood, which is obtained by integrating out the latent function values. This procedure, known as Occam's razor [49], implicitly penalises overcomplex models and is thus not prone to over-fitting. In the case of a linear kernel there are no hyper-parameters to select. The performance of the GP-based model is estimated using the same test set as the one used for the SVMs, i.e. the 16 tests corresponding to the 16 partitions used for the outer loop of the nested cross-validation scheme.

### Linear regression models

As a baseline regression method to compare results with we have built a linear model by least squares fitting (LLSF). The model has no constant term and the best fit is calculated by minimising the deviation between calculated and observed ΔΔ*G *values. It includes all 12 energy features. We have also constructed "minimal" versions of the model based on only one or two features. Model parameters are calculated on the training sets and performances evaluated on the test sets, following similar procedia employed for SVM and GP regressions.

### Measures of prediction performance

For classification, we primarily assess the prediction performances of our and related methods using the F1 score. Let *TP*, *FP*, *FN *refer to the number of true positives, false positives and false negative respectively. Precision (P, also called specificity) and recall (R, also called sensitivity) are defined as

(6)

The F1 score is the harmonic mean of precision and recall

(7)

Precision, recall and the F1 score are comprised between 0 and 1, the larger the value the better the performance. A random predictor on our data set would score *P*_*ran *_= 0.23, *R*_*ran *_= *q *and , where 0.23 is the fraction of hot spots in our database and 0 ≤ *q *≤ 1 is the fraction of presumed hot spots in the predicted set. It follows that 0 ≤ *F*1_*ran*_≤ 0.37 and if the expected frequency is equal to the observed frequency of hot spots (i.e. *R*_*ran *_= *q *= 0.23), then *F*1_*ran *_= 0.23. For each prediction set, we have also calculated the Matthew's correlation coefficient (*MCC*) given by

(8)

where *TN *is the number of true negative and *TP*, *FP *and *FN *are as above. The *MCC *value is between -1 and 1: a perfect predictor has *MCC *= 1 whereas for a random predictor *MCC *= 0 (*MCC *= -1 for a perfect inverted predictor)

The performance of the regression models is evaluated using the root-mean-square error (RMSE) and the correlation coefficient (*r*) between the predicted (*calc*) and the observed (*obs*) ΔΔ*G*

(9)

(10)

where sums are over the *N *entries in the data set and the symbols ⟨·⟩ and *σ *denote averages and standard deviations respectively. A regression model can be easily mapped into a classifier by associating a residue to an observed or predicted hot spot if ΔΔ*G *≥ 2 kcal/mol. The same performance measures used in the classification problem can then be used to assess the regression models as well.

## Authors' contributions

SL and DTJ conceived of the study; SL, MP and DTJ participated in its design; SL carried it out and drafted the manuscript, CA performed the Gaussian Processes analysis and helped to draft the manuscript. All authors revised the manuscript critically and read and approved the final version.

## Supplementary Material

Additional file 1 **Thermodynamic framework to calculate ΔΔ*G***. Theoretical calculation of ΔΔ*G *in the hypothesis of no structural changes due to binding and to alanine mutations. Click here for file

Additional file 2 **Supplementary tables and figures**. Table S1: Summary of the results obtained with SVMs using different cross-validation strategies and evaluation sets; Table S2: Correlation analysis between energy terms and the observed ΔΔ*G*; Table S3: Summary of the results for SVMs trained excluding one feature at a time; Table S4: Results of hot spot predictions for each complex in the data set; Table S5: List of protein complexes used in the investigation; Table S6: List of interacting protein domains, with the associated CATH code; Figure S1: Nested-loop cross-validation scheme; Click here for file

Additional file 3 **Data set of alanine mutations**. Data set S1: Data set of alanine mutations used in the investigation. Click here for file

Additional file 4 **The BID database**. Some considerations on the BID database, data set of alanine mutations extracted from it and summary of results obtained with our method. Click here for file

## References

[B1] Waksman G, Ed (2005). Proteomics and Protein-Protein Interactions: Biology, Chemistry, Bioinformatics, and Drug Design.

[B2] Kann MG (2007). Protein Interactions and Disease: Computational Approaches to Uncover the Etiology of Diseases. Brief Bioinform.

[B3] Arkin MR, Wells JA (2004). Small-molecule Inhibitors of Protein-protein Interactions: Progressing Towards the Dream. Nat Rev Drug Discov.

[B4] González-Ruiz D, Gohlke H (2006). Targeting Protein-protein Interactions with Small Molecules: Challenges and Perspectives for Computational Binding Epitope Detection and Ligand Finding. Curr Med Chem.

[B5] Cunningham BC, Wells JA (1989). High-resolution Epitope Mapping of hGH-receptor Interactions by Alanine-scanning Mutagenesis. Science.

[B6] Cunningham BC, Wells JA (1993). Comparison of a Structural and a Functional Epitope. J Mol Biol.

[B7] DeLano WL (2002). Unraveling hot Spots in Binding Interfaces: Progress and Challenges. Curr Opin Struct Biol.

[B8] Moreira IS, Fernandes PA, Ramos MJ (2007). Hot Spots-a Review of the Protein-protein Interface Determinant Amino-acid Residues. Proteins.

[B9] Massova I, Kollman PA (1999). Computational Alanine Scanning to Probe Protein-Protein Interactions: A Novel Approach to Evaluate Binding Free Energies. J Am Chem Soc.

[B10] Moreira IS, Fernandes PA, Ramos MJ (2007). Computational Alanine Scanning Mutagenesis-an Improved Methodological Approach. J Comput Chem.

[B11] Kortemme T, Baker D (2002). A Simple Physical Model for Binding Energy hot Spots in Protein-protein Complexes. Proc Natl Acad Sci USA.

[B12] Guerois R, Nielsen JE, Serrano L (2002). Predicting Changes in the Stability of Proteins and Protein Complexes: a Study of more than 1000 Mutations. J Mol Biol.

[B13] Darnell SJ, Page D, Mitchell JC (2007). An Automated Decision-tree Approach to Predicting Protein Interaction hot Spots. Proteins.

[B14] Bromberg Y, Rost B (2008). Comprehensive in Silico Mutagenesis Highlights Functionally Important Residues in Proteins. Bioinformatics.

[B15] Cristianini N, Shawe-Taylor J (2000). An Introduction to Support Vector Machines and Other Kernel-based Learning Methods.

[B16] Rasmussen CE, Williams CKI (2006). Gaussian Processes for Machine Learning.

[B17] Capriotti E, Fariselli P, Calabrese R, Casadio R (2005). Predicting Protein Stability Changes from Sequences Using Support Vector Machines. Bioinformatics.

[B18] Cheng J, Randall A, Baldi P (2006). Prediction of Protein Stability Changes for Single-site Mutations Using Support Vector Machines. Proteins.

[B19] Bogan AA, Thorn KS (1998). Anatomy of hot Spots in Protein Interfaces. J Mol Biol.

[B20] Noble WS (2006). What is a Support Vector Machine?. Nat Biotechnol.

[B21] Ben-Hur A, Ong CS, Sonnenburg S, Schölkopf B, Rätsch G (2008). Support Vector Machines and Kernels for Computational Biology. PLoS Comput Biol.

[B22] Baldi P, Brunak S, Chauvin Y, Andersen CA, Nielsen H (2000). Assessing the Accuracy of Prediction Algorithms for Classification: an Overview. Bioinformatics.

[B23] Li X, Keskin O, Ma B, Nussinov R, Liang J (2004). Protein-protein Interactions: hot Spots and Structurally Conserved Residues Often Locate in Complemented Pockets that Pre-organized in the Unbound States: Implications for Docking. J Mol Biol.

[B24] Keskin O, Ma B, Nussinov R (2005). Hot Regions in Protein-protein Interactions: the Organization and Contribution of Structurally Conserved hot spot Residues. J Mol Biol.

[B25] Li L, Zhao B, Cui Z, Gan J, Sakharkar MK, Kangueane P (2006). Identification of hot spot Residues at Protein-protein Interface. Bioinformation.

[B26] Cho Ki, Kim D, Lee D (2009). A Feature-based Approach to Modeling Protein-protein Interaction hot Spots. Nucleic Acids Res.

[B27] Tuncbag N, Gursoy A, Keskin O (2009). Identification of Computational hot Spots in Protein Interfaces: Combining Solvent Accessibility and Inter-residue Potentials Improves the Accuracy. Bioinformatics.

[B28] Grosdidier S, Fernandez-Recio J (2008). Identification of Hot-spot Residues in Protein-protein Interactions by Computational Docking. BMC Bioinformatics.

[B29] Shulman-Peleg A, Shatsky M, Nussinov R, Wolfson HJ (2007). Spatial Chemical Conservation of hot spot Interactions in Protein-protein Complexes. BMC Biol.

[B30] Masso M, Vaisman II (2008). Accurate Prediction of Stability Changes in Protein Mutants by Combining Machine Learning with Structure Based Computational Mutagenesis. Bioinformatics.

[B31] Ofran Y, Rost B (2007). Protein-protein Interaction Hotspots Carved into Sequences. PLoS Comput Biol.

[B32] Elcock AH (2001). Prediction of Functionally Important Residues Based Solely on the Computed Energetics of Protein Structure. J Mol Biol.

[B33] Dessailly BH, Lensink MF, Wodak SJ (2007). Relating Destabilizing Regions to Known Functional Sites in Proteins. BMC Bioinformatics.

[B34] Chen YC, Lim C (2008). Common Physical Basis of Macromolecule-binding Sites in Proteins. Nucleic Acids Res.

[B35] Kim R, Skolnick J (2008). Assessment of Programs for Ligand Binding Affinity Prediction. J Comput Chem.

[B36] Berman HM, Westbrook J, Feng Z, Gilliland G, Bhat TN, Weissig H, Shindyalov IN, Bourne PE (2000). The Protein Data Bank. Nucleic Acids Res.

[B37] Thorn KS, Bogan AA (2001). ASEdb: a Database of Alanine Mutations and Their Effects on the free Energy of Binding in Protein Interactions. Bioinformatics.

[B38] Greene LH, Lewis TE, Addou S, Cuff A, Dallman T, Dibley M, Redfern O, Pearl F, Nambudiry R, Reid A, Sillitoe I, Yeats C, Thornton JM, Orengo CA (2007). The CATH Domain Structure Database: new Protocols and Classification Levels give a more Comprehensive Resource for Exploring Evolution. Nucleic Acids Res.

[B39] Fischer TB, Arunachalam KV, Bailey D, Mangual V, Bakhru S, Russo R, Huang D, Paczkowski M, Lalchandani V, Ramachandra C, Ellison B, Galer S, Shapley J, Fuentes E, Tsai J (2003). The Binding Interface Database (BID): a Compilation of Amino acid hot Spots in Protein Interfaces. Bioinformatics.

[B40] Neria E, Fischer S, Karplus M (1996). Simulation of activation free energies in molecular systems. J Chem Phys.

[B41] Lazaridis T, Karplus M (1999). Effective Energy Function for Proteins in Solution. Proteins.

[B42] Morris G, Goodsell D, Halliday R, Huey R, Hart W, Belew R, Olson A (1998). Automated Docking Using a Lamarckian Genetic Algorithm and and Empirical Binding Free Energy Function. J Comput Chem.

[B43] McDonald IK, Thornton JM (1994). Satisfying Hydrogen Bonding Potential in Proteins. J Mol Biol.

[B44] Seok C, Rosen JB, Chodera JD, Dill KA (2003). MOPED: Method for Optimizing Physical Energy Parameters Using Decoys. J Comput Chem.

[B45] Joachims T, Schölkopf B, Burges C, Smola AJ (1999). Making large-Scale SVM Learning Practical. Advances in Kernel Methods - Support Vector Learning.

[B46] Markowetz F, Spang R (2005). Molecular Diagnosis. Classification, Model Selection and Performance Evaluation. Methods Inf Med.

[B47] Eddy SR (2004). What is Bayesian Statistics?. Nat Biotechnol.

[B48] Bishop CM (2006). Pattern Recognition and Machine Learning.

[B49] MacKay DJ (2003). Information Theory, Inference and Learning Algorithms.

