# Supplementary material for: Prediction of hot spot residues at protein-protein interfaces by combining machine learning and energy-based methods
Source: BMC Bioinformatics. 2009 Oct 30;10:365. doi: 10.1186/1471-2105-10-365 (PMC2777894; doi:10.1186/1471-2105-10-365)
Supplement: Additional file 1 — Thermodynamic framework to calculate ΔΔG. Theoretical calculation of ΔΔG in the hypothesis of no structural changes due to binding and to alanine mutations. [file 1471-2105-10-365-S1.doc]

**Additional file 1**

**Thermodynamic framework to calculate ΔΔG**

Let A and B denote the unbound monomers and AB the complex. For ease of notation we assume the alanine mutation occurs in the protein A. We further denote with (wt) the wild type molecules and with (mut) the mutated molecules. Let G be the free energy of a molecules. The binding free energy difference between the mutant and wild-type complexes is defined as

$\Delta\Delta G=\Delta G_{bind}^{(mut)}-\Delta G_{bind}^{(wt)}$, (1)

where

$\begin{matrix} \Delta G_{bind}^{(mut)}=G_{AB}^{(mut)}-G_{A}^{(mut)}-G_{B}^{(mut)} \\ \Delta G_{bind}^{(wt)}=G_{AB}^{(wt)}-G_{A}^{(wt)}-G_{B}^{(wt)} \end{matrix}$ (2)

The free energy of a molecule is G=E-TS, where E is the energy (e.g. due to electrostatic and van der Waals interactions), T is the absolute temperature and S is the entropy. Assuming constant T, it follows

$$\Delta\Delta G=\Delta\Delta E-T\Delta\Delta S\approx\Delta\Delta E$$

as the contribution of ΔΔS is usually assumed to be negligible. In molecular mechanics (MD) simulations the energy E is an average over a set of snapshots along the MD trajectory. We instead calculate E from a single conformation (e.g. the protein structure) and assume it can be decomposed as a sum of pair-wise atomic contribution

$E\equiv\sum_{i,j} e_{i,j},$ (3)

where i and j denotes the atoms. If the conformations of the two molecules A and B do not change upon binding (rigid-body docking) then from equations (2) and (3), it follows

$\begin{matrix} \Delta G_{bind}^{(mut)}=\sum_{i\in A^{(mut)},j\in B} e_{i,j} \\ \Delta G_{bind}^{(wt)}=\sum_{i\in A^{(wt)},j\in B} e_{i,j} \end{matrix}$ (4)

as the internal energy contributions of molecules A and B cancel out. In eq (4), $A^{(mut)}$ and $A^{(wt)}$represent the alanine-mutated and wild-type form of protein A respectively.

Alanine mutations eliminates side-chain atom beyond the β carbon. In the hypothesis that the position of all the other atoms do not change in the AB complex, then combining eq (1) and (4)

$\Delta\Delta G=-\sum_{i\in(sc),j\in B} e_{i,j}$ (5)

where (sc) denotes the set of side-chain atoms in the mutated amino-acid.
