# Supplementary material for: Prediction of hot spot residues at protein-protein interfaces by combining machine learning and energy-based methods
Source: BMC Bioinformatics. 2009 Oct 30;10:365. doi: 10.1186/1471-2105-10-365 (PMC2777894; doi:10.1186/1471-2105-10-365)

**Additional file 2**

**Supplementary tables and figures**

**Table S1:** Summary of results obtained with SVMs. Models differ for the cross-validation strategy (either 12 or 16 fold) and for the sets used to evaluate the performance (either test or training set). Model (a) is the main SVM model presented in the paper (16-fold cross validation, performance assessed on the test set); models (b), (c) and (d) have been built to check for potential overfitting of the data by model (a). P=precision, R=recall, F1=F1 score, MCC= Matthews correlation coefficient. When models are applied to the test set, the value of the performance measure and its associated error are calculated as described in the Material and Methods section; when models are applied to their own training sets, the values reported are the average and standard deviation over the training sets

| Model | Cross validation strategy | Evaluated on | P | R | F1 | MCC |
| --- | --- | --- | --- | --- | --- | --- |
| (a) | 16-fold | Test set | $0.53\pm0.03$ | $0.63\pm0.04$ | $0.58\pm0.02$ | $0.44\pm0.03$ |
| (b) | 12-fold | Test set | $0.51\pm0.03$ | $0.62\pm0.04$ | $0.56\pm0.03$ | $0.41\pm0.04$ |
| (c) | 16-fold | Training sets | $0.54\pm0.02$ | $0.66\pm0.03$ | $0.59\pm0.02$ | $0.46\pm0.02$ |
| (d) | 12-fold | Training sets | $0.54\pm0.02$ | $0.66\pm0.02$ | $0.59\pm0.01$ | $0.46\pm0.02$ |

**Table S2:** Correlation analysis of energy terms. The column on the left reports the values of the correlation coefficients between energy features and the observed ΔΔG. The table on the right reports correlation coefficients between pairs of energy features (values greater than 0.50 are in bold). VdW=Van der Waals, Hb=hydrogen bonds, Es=Electrostatics, Ds=Desolvation

|  |  |  |  |  | Side-chain | | | | Environment | | | |
| --- | --- | --- | --- | --- | --- | --- | --- | --- | --- | --- | --- | --- |
|  |  | ΔΔG |  |  | VdW | Hb | Es | Ds | VdW | Hb | Es | Ds |
| Side-chain | VdW | 0.47 |  |  |  | 0.38 | 0.20 | **0.79** | 0.42 | 0.27 | 0.09 | 0.31 |
|  | Hb | 0.36 |  |  |  |  | 0.39 | **0.51** | 0.24 | 0.06 | 0.06 | 0.2 |
|  | Es | 0.24 |  |  |  |  |  | 0.09 | 0.04 | 0.01 | 0.1 | 0 |
|  | Ds | 0.39 |  |  |  |  |  |  | 0.36 | 0.27 | 0.02 | 0.36 |
| Environment | VdW | 0.35 |  |  |  |  |  |  |  | **0.67** | 0.19 | **0.80** |
|  | Hb | 0.33 |  |  |  |  |  |  |  |  | 0.17 | **0.72** |
|  | Es | 0.19 |  |  |  |  |  |  |  |  |  | 0.10 |
|  | Ds | 0.33 |  |  |  |  |  |  |  |  |  |  |

**Table S3:** Summary of results for SVMs trained excluding one feature in turn (16-fold cross validation, performance assessed on test sets). P=precision, R=recall, F1=F1 score, MCC= Matthews correlation coefficient.

| Excluded feature | P | R | F1 | MCC |
| --- | --- | --- | --- | --- |
| Side-chain van der Waals | $0.49\pm0.04$ | $0.62\pm0.03$ | $0.54\pm0.02$ | $0.39\pm0.03$ |
| Side-chain hydrogen bond | $0.50\pm0.04$ | $0.58\pm0.05$ | $0.54\pm0.02$ | $0.39\pm0.03$ |
| Side-chain electrostatics | $0.53\pm0.03$ | $0.64\pm0.03$ | $0.58\pm0.02$ | $0.44\pm0.03$ |
| Side-chain desolvation | $0.49\pm0.03$ | $0.63\pm0.04$ | $0.55\pm0.02$ | $0.40\pm0.03$ |
| Environment van der Waals | $0.54\pm0.03$ | $0.63\pm0.03$ | $0.58\pm0.02$ | $0.44\pm0.02$ |
| Environment hydrogen bond | $0.54\pm0.02$ | $0.64\pm0.02$ | $0.58\pm0.02$ | $0.44\pm0.02$ |
| Environment electrostatics | $0.53\pm0.02$ | $0.62\pm0.04$ | $0.57\pm0.02$ | $0.43\pm0.03$ |
| Environment desolvation | $0.50\pm0.05$ | $0.59\pm0.05$ | $0.54\pm0.04$ | $0.39\pm0.05$ |

**Table S4:** Results of hot spot predictions for each complex. N_mut_: number of mutations, TP: true positives, TN: true negatives, FP: false positives, FN= false negatives; P: precision, R: recall, F1: F1 score, A: accuracy ( = (TP+TN)/N_mut_ ).

| PDB ID | . N_mut_ | TP | TN | FP | FN | P | R | F1 | A |
| --- | --- | --- | --- | --- | --- | --- | --- | --- | --- |
| 1A22 | 51 | 3 | 41 | 3 | 4 | 0.50 | 0.43 | 0.46 | 0.86 |
| 1A4Y | 23 | 3 | 17 | 3 | 0 | 0.50 | 1.00 | 0.67 | 0.87 |
| 1DFJ | 13 | 2 | 9 | 0 | 2 | 1.00 | 0.50 | 0.67 | 0.85 |
| 1AHW | 7 | 1 | 5 | 1 | 0 | 0.50 | 1.00 | 0.67 | 0.86 |
| 1JRH | 28 | 5 | 12 | 8 | 3 | 0.38 | 0.62 | 0.48 | 0.61 |
| 1DVF | 25 | 5 | 15 | 1 | 4 | 0.83 | 0.56 | 0.67 | 0.80 |
| 3HFM | 24 | 8 | 9 | 5 | 2 | 0.62 | 0.80 | 0.70 | 0.71 |
| 1VFB | 26 | 3 | 23 | 0 | 0 | 1.00 | 1.00 | 1.00 | 1.00 |
| 1NMB | 1 | 0 | 1 | 0 | 0 | NA | NA | NA | 1.00 |
| 1BRS | 12 | 7 | 1 | 2 | 2 | 0.78 | 0.78 | 0.78 | 0.67 |
| 1BXI | 17 | 5 | 9 | 1 | 2 | 0.83 | 0.71 | 0.77 | 0.82 |
| 1CBW | 6 | 1 | 4 | 1 | 0 | 0.50 | 1.00 | 0.67 | 0.83 |
| 2PTC | 1 | 1 | 0 | 0 | 0 | 1.00 | 1.00 | 1.00 | 1.00 |
| 1DAN | 53 | 2 | 43 | 8 | 0 | 0.20 | 1.00 | 0.33 | 0.85 |
| 1DX5 | 15 | 1 | 9 | 1 | 4 | 0.50 | 0.20 | 0.29 | 0.67 |
| 1FC2 | 3 | 0 | 2 | 0 | 1 | NA | 0.00 | NA | 0.67 |
| 1FCC | 8 | 4 | 3 | 1 | 0 | 0.80 | 1.00 | 0.89 | 0.88 |
| 1JCK | 9 | 0 | 5 | 0 | 4 | NA | 0.00 | NA | 0.56 |
| 1GC1 | 17 | 0 | 15 | 2 | 0 | 0 | NA | NA | 0.88 |
| 1JTG | 10 | 2 | 3 | 5 | 0 | 0.29 | 1.00 | 0.44 | 0.50 |

**Table S5**: List of protein complexes used in the investigation. Within parenthesis, the number of hot spots in each protein.

| PDB  ID | Protein 1 | Protein 2 | Chain  protein 1 | Chain  protein 2 | Number of  mutations  protein 1 | Number of  mutations  protein 2 | Data  source |
| --- | --- | --- | --- | --- | --- | --- | --- |
| 1A22 | hGH | hGHbp | A | B | 24 (3) | 27 (4) | [1,2] |
| 1A4Y | RNase inhibitor | Angiogenin | A | B | 12 (2) | 11 (1) | ASEdb |
| 1DFJ | RNase inhibitor | RNase A | I | E | 13 (4) | 0 (0) | ASEdb |
| 1AHW | Fab 5G9 | Tissue factor | AB | C | 0 (0) | 7 (1) | ASEdb |
| 1JRH | Antibody A6 | Interferon-γ   receptor | LH | I | 17 (3) | 11 (5) | [3,4] |
| 1DVF | FV D1.3 | FV E5.2 | AB | CD | 16 (6) | 9 (3) | ASEdb |
| 3HFM | HYHEL-10 | HEL | LH | Y | 11 (7) | 13 (3) | ASEdb, [5] |
| 1VFB | D1.3 | HEL | AB | C | 14 (2) | 12 (1) | ASEdb |
| 1NMB | N9 Neuraminidase | Fab NC10 | N | LH | 0 (0) | 1 (0) | [6] |
| 1BRS | Barnase | Barstar | A | D | 7 (6) | 5 (3) | ASEdb |
| 1BXI | Im9 | E9 DNase | A | B | 17 (7) | 0 (0) | ASEdb |
| 1CBW | Cymotrypsin | BPTI | ABC | D | 0 (0) | 6 (1) | ASEdb |
| 2PTC | Trypsin | BPTI | E | I | 0 (0) | 1 (1) | ASEdb |
| 1DAN | Factor VIIA | Tissue factor | LH | TU | 23 (0) | 30 (2) | ASEdb |
| 1DX5 | Thrombin | Thrombomodulin | M | I | 15 (5) | 0 (0) | [7] |
| 1FC2 | Protein A | IgG1 | C | D | 3 (1) | 0 (0) | ASEdb |
| 1FCC | protein G | IGG | C | A | 8 (4) | 0 (0) | [8] |
| 1JCK | T-cell antigen receptor | SEC3-1A4 | A | B | 0 (0) | 9 (4) | ASEdb |
| 1GC1 | CD4 | Envelope protein GP120 | C | G | 17 (0) | 0 (0) | ASEdb |
| 1JTG | TEM-1 β-lactamase | BLIP | A | B | 6 (0) | 4 (2) | [9] |

ASEdb: http://nic.ucsf.edu/asedb/

[1] BC Cunningham, and JA Wells, J Mol Biol **234,** 554 (1993)

[2] T Clackson, M Ultsch, JA Wells, and AM de Vos, J Mol Biol **277,** 1111 (1998)

[3] K Hofstadter et al, J Mol Biol **285,** 805 (1999)

[4] S Lang et al., Biochemistry **39**, 15674 (2000)

[5] J Pons et al, Protein Sci **8**, 958 (1999)

[6] DA Dougan et al, Protein Eng **11**, 65 (1998)

[7] AO Pineda et al, J Biol Chem **277**, 32015 (2002)

[8] DJ Sloan and HW Hellinga, Protein Sci **8**, 1643 (1999)

[9] D Reichmann et al, Proc Natl Acad Sci **102**, 57 (2005)

**Table S6**: List of interacting protein domains, with the associated CATH codes. Note that 10 complexes out of 20 contain an immunoglobin domain (code 2.60.40.10). Horizontal lines group complexes for the 16-fold cross-validation. Brackets on the right side of the table cluster some structures even further and produce a 12-fold partition of the dataset.

| PDB  ID | Interacting  domain(s)  protein 1 | Interacting  domain(s)  protein 1 | CATH code(s)  protein 1 | CATH code(s)  protein 1 |
| --- | --- | --- | --- | --- |
| 1A22 | A00 | B01-B02 | 1.20.1250.10.12 | 2.60.40.30.26 - 2.60.40.30.12 |
| 1A4Y ^(c)^ | A00 | B00 | 3.80.10.10.13 | 3.10.130.10.5 |
| 1DFJ ^(c)^ | I00 | E00 | 3.80.10.10.13 | 3.10.130.10.1 |
| 1AHW | A01-B01 | C02 | 2.60.40.10.4 - 2.60.40.10.6 | 2.60.40.30.1 |
| 1JRH | L01-H01 | I00 | 2.60.40.10.4 - 2.60.40.10.23 | 2.60.40.30.9 |
| 1DVF | A00-B00 | C00-D00 | 2.60.40.10.4 - 2.60.40.10.23 | 2.60.40.10.4 - 2.60.40.10.6 |
| 3HFM ^(a)^ | L01-H01 | Y00 | 2.60.40.10.3 - 2.60.40.10.23 | 1.10.530.10.1 |
| 1VFB ^(a)^ | A00-B00 | C00 | 2.60.40.10.4 - 2.60.40.10 23 | 1.10.530.10.1 |
| 1NMB | N00 | L00-H00 | 2.120.10.10.2 | 2.60.40.10.6 - 2.60.40.10.4 |
| 1BRS | A00 | D00 | 3.10.450.30.3 | 3.30.370.10.1 |
| 1BXI | A00 | B00 | 1.10.1200.20.1 | 3.90.540.10.1 |
| 1CBW ^(c)^ | B00-C00 | D00 | 2.40.10.10.35 - 2.40.10.10.29 | 4.10.410.10.1 |
| 2PTC ^(c)^ | E01-E02 | I00 | 2.40.10.10.5 - 2.40.10.10.7 | 4.10.410.10.1 |
| 1DAN ^(b)^ | L??-H01 | T00-U00 | ?.?.?.?.? – 2.40.10.10.3 | 2.60.40.30.24 - 2.60.40.30.1 |
| 1DX5 | M01 | I02-I03 | 2.40.10.10.11 | 2.10.25.10.26 - 2.10.25.10.19 |
| 1FC2 | C00 | D01-D02 | 1.20.5.420.1 | 2.60.40.10.29 - 2.60.40.10.18 |
| 1FCC | C00 | A01-A02 | 3.10.20.10.1 | 2.60.40.10.29 - 2.60.40.10.18 |
| 1JCK | A01 | B01-B02 | 2.60.40.10.12 | 2.40.50.110.2 - 3.10.20.120.1 |
| 1GC1 | C01-C02 | G00 | 2.60.40.10.65 - 2.60.40.10.128 | 2.170.40.20.1 |
| 1JTG | A00 | B01-B02 | 3.40.710.10.1 | 3.30.1450.10.2 - 3.30.1450.10.1 |

(a) 3HFM and 1VFB share a pair of similar (at the S-level) interacting domains. They are nonetheless both included in the database because the interaction interface is not the same. 3HFM:Y00 and 1VFB:C00 are 100% identical but their interface has only 4 residues (out of 22) in common. After optimal structural superposition (using the SSAP server), we have verified that 3HFM and 1VFB have no equivalent residue contacts.

(b) 1DANL has not yet been classified in CATH but a search with the CATHREDAL server does not return any obvious similarity with other entries in CATH, nor do SSAP structural alignments with the other domains in the dataset. 1DAN:U00 is 96% identical to 1AHW:C02 but they use a different binding interface with only 4/27 residues in common. For this reason we have kept them separated when clustering with the more stringent criteria that group similar domains, independently of their binding partners.

(c) A small number of mutations is at equivalent positions in the two homologous complexes 1A4Y-1DFJ and 1CBW-2PTC, respectively 10 and 1 mutations. For the complexes 1A4Y-1DFJ these are mutations respectively at residues A:261-I:257, A:263-I:259, A:289-I:285, A:318-I:314, A:320-I:316, A:401-I:397, A:434-I:430, A:435-I:431, A:437-I:433 and A:459-I:455; for the complexes 1CBW-2PTC it is the mutation respectively at D:15-I:15 (the notation stands for “chain”:”residue number”) .

**Figure S1**: Nested-loop cross validation scheme. An outer n-fold cross validation produces n training sets (circled in blue) and n testing sets (in red). An inner (n-1)-fold cross validation is applied to each training set to choose the hyper-parameters. The best performing model is then applied to the corresponding test set. The model is also applied to its own training set as a check for data over-fitting. Predictions on the test sets are then gathered in order to assess the performance on the whole dataset


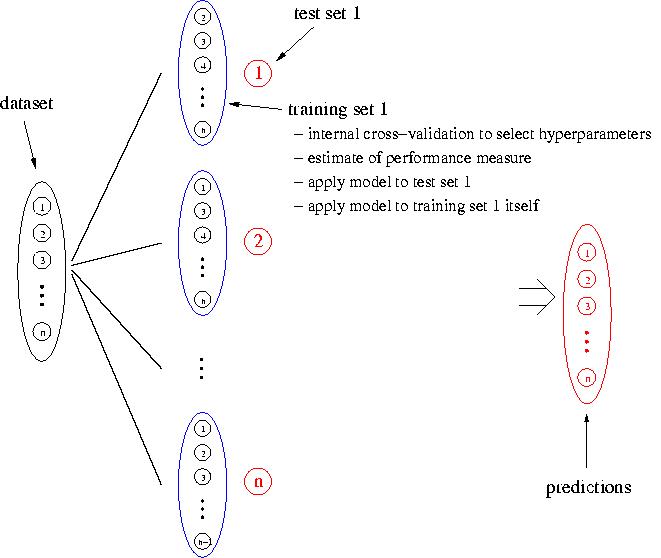

Supplement: Additional file 2 — Supplementary tables and figures. Table S1: Summary of the results obtained with SVMs using different cross-validation strategies and evaluation sets; Table S2: Correlation analysis between energy terms and the observed ΔΔG; Table S3: Summary of the results for SVMs trained excluding one feature at a time; Table S4: Results of hot spot predictions for each complex in the data set; Table S5: List of protein complexes used in the investigation; Table S6: List of interacting protein domains, with the associated CATH code; Figure S1: Nested-loop cross-validation scheme; [file 1471-2105-10-365-S2.doc]
