# Supplementary material for: Prediction of hot spot residues at protein-protein interfaces by combining machine learning and energy-based methods
Source: BMC Bioinformatics. 2009 Oct 30;10:365. doi: 10.1186/1471-2105-10-365 (PMC2777894; doi:10.1186/1471-2105-10-365)
Supplement: Additional file 3 — Data set of alanine mutations. Data set S1: Data set of alanine mutations used in the investigation. [file 1471-2105-10-365-S3.doc]

**Additional file 3**

**Data set of alanine mutations**

**Data set S1:** Data set of alanine mutations used in the investigation. We report both the measured ΔΔG and the prediction from Robetta. Positive scores for SVM and TSVM are residues predicted to have ΔΔG ≥ 2 kcal/mol

| **Pdb id** | **Chain** | **PDB residue number** | **Residue type** | **Observed ΔΔG (kcal/mol)** | **Robetta ΔΔG (kcal/mol)** | **SVM score** | **TSVM score** |
| --- | --- | --- | --- | --- | --- | --- | --- |
| 1A22 | A | 18 | H | -0.5 | 2.29 | -0.61 | -0.75 |
| 1A22 | A | 21 | H | 0.2 | 0.83 | -0.3 | -0.64 |
| 1A22 | A | 22 | Q | -0.2 | 0.05 | -1.1 | -1.57 |
| 1A22 | A | 25 | F | -0.4 | 1.29 | -0.61 | -0.92 |
| 1A22 | A | 42 | Y | 0.2 | 2.02 | 0 | -0.1 |
| 1A22 | A | 45 | L | 1.2 | 1.15 | -0.28 | -0.66 |
| 1A22 | A | 46 | Q | 0.1 | 1.04 | -0.5 | -0.51 |
| 1A22 | A | 51 | S | 0.3 | -0.02 | -0.78 | -1.31 |
| 1A22 | A | 56 | E | 0.4 | 0.97 | -0.74 | -0.96 |
| 1A22 | A | 62 | S | 0.1 | -0.17 | -0.5 | -1.13 |
| 1A22 | A | 63 | N | 0.3 | 0.4 | -0.68 | -1.25 |
| 1A22 | A | 64 | R | 1.6 | 2 | 0.81 | 1.11 |
| 1A22 | A | 65 | E | -0.5 | -0.11 | -0.72 | -1.24 |
| 1A22 | A | 68 | Q | 0.6 | 1.81 | -0.07 | 0 |
| 1A22 | A | 164 | Y | 0.3 | 0.99 | -0.84 | -1.22 |
| 1A22 | A | 167 | R | 0.3 | 0.42 | -0.44 | -0.66 |
| 1A22 | A | 168 | K | -0.2 | 2.12 | -0.38 | -0.57 |
| 1A22 | A | 171 | D | 0.8 | 9 | 0.39 | 0.2 |
| 1A22 | A | 172 | K | 2 | 0.74 | -0.58 | -0.95 |
| 1A22 | A | 174 | E | -0.9 | 1.38 | -0.32 | -0.77 |
| 1A22 | A | 175 | T | 2 | 2.31 | 0.11 | -0.19 |
| 1A22 | A | 176 | F | 1.9 | 0.61 | -0.84 | -1.3 |
| 1A22 | A | 178 | R | 2.4 | 0.38 | -0.37 | -0.76 |
| 1A22 | A | 179 | I | 0.8 | 1 | -0.28 | -0.67 |
| 1A22 | B | 243 | R | 2.12 | 6.54 | 0.33 | 0.37 |
| 1A22 | B | 244 | E | 1.69 | 0.49 | -0.29 | -0.59 |
| 1A22 | B | 271 | R | 0.54 | 1.31 | -0.07 | -0.12 |
| 1A22 | B | 273 | T | 0.11 | 0 | -1.37 | -1.91 |
| 1A22 | B | 274 | Q | 0 | 0.03 | -1.36 | -1.91 |
| 1A22 | B | 275 | E | -0.1 | 0.09 | -1.19 | -1.69 |
| 1A22 | B | 276 | W | 0.51 | 2.86 | -0.39 | -0.28 |
| 1A22 | B | 298 | S | -0.05 | 0.29 | -1.28 | -1.9 |
| 1A22 | B | 301 | T | 1.76 | 0.02 | -1.13 | -1.72 |
| 1A22 | B | 302 | S | -0.2 | -0.11 | -0.9 | -1.49 |
| 1A22 | B | 303 | I | 1.61 | 0.31 | -0.12 | -0.75 |
| 1A22 | B | 304 | W | >4.50 | 5.38 | 1.25 | 1.61 |
| 1A22 | B | 305 | I | 1.94 | 0.13 | -0.68 | -1.17 |
| 1A22 | B | 320 | E | -0.19 | 0.67 | -0.69 | -0.72 |
| 1A22 | B | 321 | K | 0.08 | 0.05 | -0.98 | -1.57 |
| 1A22 | B | 324 | S | 0.28 | -0.06 | -0.45 | -1.07 |
| 1A22 | B | 326 | D | 0.99 | -0.31 | -0.08 | -0.6 |
| 1A22 | B | 327 | E | 0.97 | 1.11 | -0.1 | -0.04 |
| 1A22 | B | 364 | D | 1.49 | 1.37 | -0.09 | 0.15 |
| 1A22 | B | 365 | I | 2.13 | 0.09 | -1.05 | -1.49 |
| 1A22 | B | 366 | Q | 0.02 | 0.68 | -0.94 | -1.49 |
| 1A22 | B | 367 | K | -0.02 | 0.22 | -0.61 | -1.18 |
| 1A22 | B | 369 | W | >4.50 | 3.56 | 0.73 | 0.83 |
| 1A22 | B | 371 | V | -0.64 | 0.26 | -0.76 | -1.22 |
| 1A22 | B | 417 | R | 0.28 | 0.22 | -0.37 | -0.65 |
| 1A22 | B | 418 | N | 0.3 | 1.84 | 0.1 | -0.11 |
| 1A22 | B | 419 | S | 0.03 | 0.98 | -0.3 | -0.93 |
| 1A4Y | A | 261 | W | 0.1 | 1.06 | -0.9 | -1.25 |
| 1A4Y | A | 263 | W | 1.2 | 2.27 | -0.21 | -0.21 |
| 1A4Y | A | 289 | S | 0 | 0.6 | -0.9 | -1.71 |
| 1A4Y | A | 318 | W | 1.5 | 2.19 | -0.4 | -0.55 |
| 1A4Y | A | 320 | K | -0.3 | -0.21 | -1.06 | -1.76 |
| 1A4Y | A | 344 | E | 0.2 | 1.37 | -0.86 | -1.33 |
| 1A4Y | A | 375 | W | 1 | 2.83 | -0.76 | -0.8 |
| 1A4Y | A | 401 | E | 0.9 | 0.02 | -1.18 | -1.72 |
| 1A4Y | A | 434 | Y | 3.3 | 3 | 0.21 | 0.23 |
| 1A4Y | A | 435 | D | 3.5 | 0.57 | 0.39 | 0.54 |
| 1A4Y | A | 437 | Y | 0.8 | 3.13 | 0.03 | 0.32 |
| 1A4Y | A | 459 | I | 0.7 | 0.62 | -1.13 | -1.77 |
| 1A4Y | B | 5 | R | 2.3 | 2.54 | 0.02 | 0.68 |
| 1A4Y | B | 8 | H | 0.9 | 0.85 | -1.03 | -1.43 |
| 1A4Y | B | 12 | Q | 0.3 | 0.87 | -0.55 | -1.06 |
| 1A4Y | B | 13 | H | -0.3 | 0.02 | -1.22 | -1.83 |
| 1A4Y | B | 31 | R | 0.2 | 2.76 | 0.24 | 1.03 |
| 1A4Y | B | 32 | R | 0.9 | 0.18 | -0.56 | -0.52 |
| 1A4Y | B | 68 | N | 0.2 | 0.52 | -1.33 | -2.21 |
| 1A4Y | B | 84 | H | 0.2 | 1.05 | -0.52 | -0.95 |
| 1A4Y | B | 89 | W | 0.2 | 2.71 | -0.06 | 0.2 |
| 1A4Y | B | 108 | E | -0.3 | 1.73 | -0.42 | -0.66 |
| 1A4Y | B | 114 | H | 0.65 | 1.69 | -0.29 | -0.73 |
| 1DFJ | I | 202 | E | 1 | 0.77 | -0.72 | -1.01 |
| 1DFJ | I | 257 | W | 1.3 | 1.58 | -0.74 | -1.07 |
| 1DFJ | I | 259 | W | 2.2 | 3.09 | -0.4 | -0.44 |
| 1DFJ | I | 283 | E | 1.3 | -0.1 | -1.37 | -1.77 |
| 1DFJ | I | 285 | S | 0.8 | 0 | -1.22 | -2.14 |
| 1DFJ | I | 314 | W | 1 | 1.27 | -0.98 | -1.17 |
| 1DFJ | I | 316 | K | 1.3 | 0.38 | -1.17 | -1.56 |
| 1DFJ | I | 397 | E | 1.3 | 0.42 | -1.15 | -1.39 |
| 1DFJ | I | 430 | Y | 5.9 | 2.72 | 0.08 | 0.31 |
| 1DFJ | I | 431 | D | 3.6 | 0.16 | -0.33 | -0.68 |
| 1DFJ | I | 433 | Y | 2.6 | 3.62 | 1.08 | 2.01 |
| 1DFJ | I | 453 | R | 0.8 | 0.21 | -1.16 | -1.45 |
| 1DFJ | I | 455 | I | 0.3 | 0.5 | -0.77 | -1.43 |
| 1AHW | C | 156 | Y | >4.00 | 4.53 | 0.66 | 0.97 |
| 1AHW | C | 167 | T | 0 | -0.24 | 0.8 | 0.96 |
| 1AHW | C | 170 | T | 1 | -0.06 | -0.69 | -0.81 |
| 1AHW | C | 178 | D | -0.5 | -0.09 | -1.6 | -1.95 |
| 1AHW | C | 197 | T | 1.3 | -0.01 | -1.15 | -1.4 |
| 1AHW | C | 198 | V | -0.3 | -0.01 | -1.19 | -1.42 |
| 1AHW | C | 199 | N | 1.1 | -0.01 | -1.34 | -1.61 |
| 1JRH | L | 27 | E | 0.54 | 0.67 | -1.02 | -1.31 |
| 1JRH | L | 28 | D | 0.44 | 0.67 | -1.23 | -1.54 |
| 1JRH | L | 30 | Y | 1.1 | 1.09 | -0.39 | -0.44 |
| 1JRH | L | 91 | Y | 0.58 | 0.28 | 0.32 | 0.51 |
| 1JRH | L | 92 | W | 2.8 | 3.01 | 1.01 | 1.21 |
| 1JRH | L | 93 | S | -0.65 | 0.54 | 0.6 | 0.77 |
| 1JRH | L | 94 | T | 0.38 | 0.36 | 0 | -0.06 |
| 1JRH | L | 96 | W | 1.7 | 0.59 | -0.81 | -1.1 |
| 1JRH | H | 32 | Y | 1.4 | 1.63 | -0.77 | -0.88 |
| 1JRH | H | 52 | W | 2.7 | 1.55 | -0.23 | -0.37 |
| 1JRH | H | 53 | W | 2.4 | 0.73 | -1.04 | -1.33 |
| 1JRH | H | 54 | D | 1.9 | 1.64 | -0.84 | -1.08 |
| 1JRH | H | 56 | D | 1.8 | 0.49 | -0.7 | -0.88 |
| 1JRH | H | 58 | Y | 1.2 | 2.05 | 0.2 | 0.29 |
| 1JRH | H | 95 | R | 0.54 | 1.49 | 0.08 | 0.16 |
| 1JRH | H | 99 | Y | 1.1 | 1.98 | 0.76 | 1.16 |
| 1JRH | H | 100B | H | 1.7 | 3.38 | 1.9 | 2.44 |
| 1JRH | I | 47 | K | 3.6 | 1.4 | 0.1 | 0.06 |
| 1JRH | I | 49 | Y | 3.4 | 3.87 | 2.91 | 3.79 |
| 1JRH | I | 51 | V | 1.9 | 1 | 0.78 | 1 |
| 1JRH | I | 52 | K | 3 | 2.38 | 2.37 | 2.71 |
| 1JRH | I | 53 | N | 3.9 | 2.72 | 2.3 | 2.87 |
| 1JRH | I | 54 | S | 0.3 | -0.03 | 0.39 | 0.37 |
| 1JRH | I | 55 | E | -0.4 | -0.1 | -0.13 | -0.24 |
| 1JRH | I | 79 | N | -0.4 | 0.03 | -0.21 | -0.17 |
| 1JRH | I | 82 | W | 4.5 | 1.35 | -0.84 | -0.98 |
| 1JRH | I | 84 | R | -0.3 | 0.37 | -1.19 | -1.48 |
| 1JRH | I | 98 | K | 0 | 0.05 | -1 | -1.28 |
| 1DVF | A | 30 | H | 1.7 | 0.26 | -1.34 | -1.4 |
| 1DVF | A | 32 | Y | 2 | 0.47 | -0.72 | -0.77 |
| 1DVF | A | 49 | Y | 1.7 | 0.88 | -1.44 | -1.36 |
| 1DVF | A | 50 | Y | 0.7 | 0.58 | -1.06 | -1.32 |
| 1DVF | A | 92 | W | 0.3 | 1.56 | -0.44 | -0.75 |
| 1DVF | A | 93 | S | 1.2 | -0.02 | -1.55 | -1.59 |
| 1DVF | B | 30 | T | 0.9 | 0.01 | -1.24 | -1.34 |
| 1DVF | B | 32 | Y | 1.8 | 0.11 | -0.57 | -0.87 |
| 1DVF | B | 52 | W | 4.2 | 3.26 | 1.65 | 1.35 |
| 1DVF | B | 54 | D | 4.3 | 0.72 | 0.82 | 0.81 |
| 1DVF | B | 56 | N | 1.2 | 1.07 | -0.1 | -0.36 |
| 1DVF | B | 58 | D | 1.6 | 0.71 | -0.78 | -0.78 |
| 1DVF | B | 98 | E | 4.2 | 0.05 | -0.72 | -0.54 |
| 1DVF | B | 99 | R | 1.9 | 0.22 | -0.41 | -0.4 |
| 1DVF | B | 100 | D | 2.8 | 0.43 | -0.43 | -0.5 |
| 1DVF | B | 101 | Y | >4.00 | 2.13 | 0.18 | 0.44 |
| 1DVF | C | 49 | Y | 1.9 | 0.3 | -0.96 | -0.86 |
| 1DVF | D | 30 | K | 1 | -0.04 | -1.76 | -1.87 |
| 1DVF | D | 33 | H | 1.9 | 0.79 | -1.39 | -1.47 |
| 1DVF | D | 52 | D | 1.7 | -0.23 | -1.18 | -1.27 |
| 1DVF | D | 54 | N | 1.9 | 2.09 | -1.31 | -1.2 |
| 1DVF | D | 97 | I | 2.7 | 1.07 | -0.51 | -0.69 |
| 1DVF | D | 98 | Y | 4.7 | 4.78 | 2.75 | 2.74 |
| 1DVF | D | 100 | Q | 1.6 | 1.44 | 0.68 | 1.07 |
| 1DVF | D | 100B | R | 4.1 | 1.69 | 0.27 | 0.52 |
| 3HFM | L | 31 | N | 5.2 | 1.86 | 0.17 | 0.13 |
| 3HFM | L | 32 | N | 5.13 | 2.48 | -0.19 | -0.16 |
| 3HFM | L | 50 | Y | 4.6 | 1.4 | 0.39 | 0.24 |
| 3HFM | L | 53 | Q | 0.96 | 0.83 | -0.54 | -0.29 |
| 3HFM | L | 96 | Y | 2.73 | 0.45 | -0.57 | -0.76 |
| 3HFM | H | 31 | S | 0.18 | 0.25 | -0.85 | -0.59 |
| 3HFM | H | 32 | D | 1.93 | 1.1 | -0.97 | -1.21 |
| 3HFM | H | 33 | Y | 6 | 2.9 | 1.31 | 1.72 |
| 3HFM | H | 50 | Y | 7.4 | 2.96 | -0.17 | 0.42 |
| 3HFM | H | 53 | Y | 3.3 | 1.66 | 0.61 | 0.88 |
| 3HFM | H | 58 | Y | 1.7 | 1.77 | 0.3 | 0.33 |
| 3HFM | Y | 15 | H | -0.44 | 0.09 | -0.56 | -0.88 |
| 3HFM | Y | 20 | Y | 4.9 | 2.23 | 0.19 | 0.12 |
| 3HFM | Y | 21 | R | 1.07 | 3.42 | 1.16 | 1.92 |
| 3HFM | Y | 63 | W | 0.31 | 0.83 | -0.99 | -1.21 |
| 3HFM | Y | 73 | R | -0.33 | 0.56 | -0.65 | -0.66 |
| 3HFM | Y | 75 | L | 0.69 | 1.3 | -0.79 | -0.55 |
| 3HFM | Y | 89 | T | 0 | 0.17 | -0.88 | -1.22 |
| 3HFM | Y | 93 | N | 0.21 | 1.53 | -0.41 | 0 |
| 3HFM | Y | 96 | K | 7 | 2.13 | 0.75 | 0.99 |
| 3HFM | Y | 97 | K | 6.2 | 1.42 | -0.18 | 0.2 |
| 3HFM | Y | 98 | I | 0 | 0.1 | -1.1 | -1.06 |
| 3HFM | Y | 100 | S | 0.26 | 0.84 | 0.38 | 0.62 |
| 3HFM | Y | 101 | D | 0.94 | 0.32 | 1.4 | 1.49 |
| 1VFB | A | 30 | H | 0.8 | 0.26 | -0.92 | -1.16 |
| 1VFB | A | 32 | Y | 1.3 | 1.31 | -0.18 | -0.3 |
| 1VFB | A | 49 | Y | 0.8 | 0.49 | -1.02 | -1.24 |
| 1VFB | A | 50 | Y | 0.4 | 1.52 | -0.09 | -0.14 |
| 1VFB | A | 53 | T | -0.23 | 0.64 | -1.29 | -1.32 |
| 1VFB | A | 92 | W | 1.71 | 2.06 | -0.49 | -0.6 |
| 1VFB | A | 93 | S | 0.11 | 0 | -0.74 | -1.07 |
| 1VFB | B | 30 | T | 0.09 | 0.66 | -1.44 | -1.81 |
| 1VFB | B | 32 | Y | 0.5 | 0.66 | -1.18 | -1.52 |
| 1VFB | B | 52 | W | 1.23 | 1.75 | -0.29 | -0.41 |
| 1VFB | B | 54 | D | 1.95 | -0.14 | -1.01 | -1.32 |
| 1VFB | B | 99 | R | 0.47 | 0.73 | -0.66 | -0.72 |
| 1VFB | B | 100 | D | 3.1 | 3.03 | 0.71 | 1.09 |
| 1VFB | B | 101 | Y | >4.00 | 3.23 | 1.15 | 1.34 |
| 1VFB | C | 18 | D | 0.3 | 0.55 | -0.96 | -1.11 |
| 1VFB | C | 19 | N | 0.3 | 1 | -0.27 | -0.25 |
| 1VFB | C | 23 | Y | 0.4 | 0 | -0.66 | -0.85 |
| 1VFB | C | 24 | S | 0.8 | 0.71 | -0.03 | -0.09 |
| 1VFB | C | 116 | K | 0.7 | 0.85 | -0.74 | -1.07 |
| 1VFB | C | 118 | T | 0.8 | 0.1 | -0.59 | -0.89 |
| 1VFB | C | 119 | D | 1 | 1.67 | 0.02 | -0.12 |
| 1VFB | C | 120 | V | 0.9 | 0.23 | -0.41 | -0.57 |
| 1VFB | C | 121 | Q | 2.9 | 4.2 | 0.92 | 1.38 |
| 1VFB | C | 124 | I | 1.2 | 0.43 | -1.05 | -1.17 |
| 1VFB | C | 125 | R | 1.8 | 2.22 | -0.38 | -0.57 |
| 1VFB | C | 129 | L | 0.2 | 0.06 | -1.41 | -1.7 |
| 1NMB | H | 99 | Y | 1.5 | 1.28 | -0.59 | -0.9 |
| 1BRS | A | 27 | K | 5.4 | 1.88 | -0.01 | 0.04 |
| 1BRS | A | 58 | N | 3.1 | -0.03 | -1.03 | -1.53 |
| 1BRS | A | 59 | R | 5.2 | 3.01 | 1.15 | 1.33 |
| 1BRS | A | 60 | E | -0.2 | 1.41 | 0.24 | 0.13 |
| 1BRS | A | 73 | E | 2.8 | -0.2 | -0.36 | -0.63 |
| 1BRS | A | 87 | R | 5.5 | 4.44 | 0.2 | 0.25 |
| 1BRS | A | 102 | H | 6 | 5.08 | 2.02 | 2.47 |
| 1BRS | D | 29 | Y | 3.4 | 3.13 | 1.05 | 1.4 |
| 1BRS | D | 35 | D | 4.5 | 1.42 | 2.44 | 2.87 |
| 1BRS | D | 39 | D | 7.7 | 9.4 | 2.94 | 3.87 |
| 1BRS | D | 42 | T | 1.8 | 1.66 | 0.44 | 0.44 |
| 1BRS | D | 76 | E | 1.3 | 1.54 | -0.81 | -0.92 |
| 1BXI | A | 23 | C | 0.92 | -0.08 | -0.44 | -0.59 |
| 1BXI | A | 24 | N | 0.14 | 0 | -1.03 | -1.47 |
| 1BXI | A | 27 | T | 0.73 | 0.6 | -0.75 | -0.84 |
| 1BXI | A | 28 | S | 0.17 | 0 | -1.19 | -1.55 |
| 1BXI | A | 29 | S | 0.96 | 0.17 | -0.81 | -1.12 |
| 1BXI | A | 30 | E | 1.41 | 2.97 | 1.51 | 1.93 |
| 1BXI | A | 33 | L | 3.42 | 1.02 | -0.23 | -0.64 |
| 1BXI | A | 34 | V | 2.58 | 0.98 | 0.39 | 0.16 |
| 1BXI | A | 37 | V | 1.66 | 0.5 | -0.52 | -0.91 |
| 1BXI | A | 38 | T | 0.9 | 1.35 | -0.55 | -0.77 |
| 1BXI | A | 41 | E | 2.08 | -0.07 | -0.08 | -0.47 |
| 1BXI | A | 48 | S | 0.01 | -0.01 | -0.9 | -1.32 |
| 1BXI | A | 50 | S | 2.19 | 4.21 | 0.69 | 1.02 |
| 1BXI | A | 51 | D | 5.92 | 0.82 | 0.56 | 0.48 |
| 1BXI | A | 53 | I | 0.85 | 0.17 | 0.08 | -0.22 |
| 1BXI | A | 54 | Y | 4.83 | 2.85 | 2.15 | 2.24 |
| 1BXI | A | 55 | Y | 4.63 | 3.49 | 1.61 | 1.7 |
| 1CBW | D | 11 | T | 0.2 | 0.18 | -1 | -1.37 |
| 1CBW | D | 15 | K | 2 | 1.58 | 1.23 | 1.02 |
| 1CBW | D | 17 | R | 0.5 | 1.53 | 0.87 | 0.68 |
| 1CBW | D | 19 | I | 0.1 | 0.68 | -1.17 | -1.58 |
| 1CBW | D | 34 | V | 0 | 0.3 | -0.8 | -1.24 |
| 1CBW | D | 39 | R | 0.2 | 1.58 | -0.92 | -1.22 |
| 2PTC | I | 15 | K | 10 | 4.16 | 2.33 | 2.55 |
| 1DAN | L | 39 | L | 0 | 1.24 | -0.64 | -0.93 |
| 1DAN | L | 62 | K | 0 | -0.27 | -0.31 | -0.53 |
| 1DAN | L | 64 | Q | 0.8 | 2.98 | -0.24 | -0.05 |
| 1DAN | L | 69 | I | 1.9 | 1.57 | -0.31 | -0.7 |
| 1DAN | L | 71 | F | 1.2 | 3.07 | -0.03 | 0.03 |
| 1DAN | L | 73 | L | 0 | 0 | -1.37 | -1.76 |
| 1DAN | L | 77 | E | 0 | 0.36 | 0.41 | 0.47 |
| 1DAN | L | 79 | R | 1.2 | 2.45 | 0.4 | 0.77 |
| 1DAN | L | 88 | Q | 0 | 0.35 | -0.87 | -1.28 |
| 1DAN | L | 92 | V | 0 | 1.05 | -0.98 | -1.31 |
| 1DAN | L | 93 | N | 0 | 0.12 | -1.09 | -1.41 |
| 1DAN | L | 94 | E | 0 | 0.02 | -1.1 | -1.42 |
| 1DAN | H | 129F | F | 0 | 1.66 | 0.14 | -0.12 |
| 1DAN | H | 134 | R | 0.51 | 3.09 | 0.55 | 0.69 |
| 1DAN | H | 135 | F | 0 | 0.53 | -0.82 | -1.29 |
| 1DAN | H | 162 | R | 0.65 | -0.06 | -0.43 | -0.96 |
| 1DAN | H | 163 | L | 0.7 | 0 | -0.59 | -1.11 |
| 1DAN | H | 164 | M | 0.5 | 1.03 | 0.43 | 0.09 |
| 1DAN | H | 165 | T | 0 | 0.27 | -0.62 | -1.19 |
| 1DAN | H | 166 | Q | 0 | 2.6 | 0.37 | 0.16 |
| 1DAN | H | 167 | D | 0.41 | 1.34 | 0.22 | 0.19 |
| 1DAN | H | 170 | Q | 0 | 0 | -0.83 | -1.33 |
| 1DAN | H | 230 | R | 0.51 | 1.33 | -0.87 | -0.9 |
| 1DAN | T | 17 | T | 0.1 | 0.12 | -0.95 | -1.45 |
| 1DAN | T | 18 | N | 0.2 | 0.04 | -0.85 | -1.39 |
| 1DAN | T | 20 | K | 2.6 | 1.5 | 0.03 | 0.16 |
| 1DAN | T | 22 | I | 0.7 | 0.65 | -0.7 | -1.06 |
| 1DAN | T | 24 | E | 0.7 | 0.64 | -0.97 | -1.16 |
| 1DAN | T | 37 | Q | 0.55 | 1.41 | -0.69 | -1.08 |
| 1DAN | T | 41 | K | 0.35 | -0.04 | -1.3 | -1.77 |
| 1DAN | T | 42 | S | -0.1 | -0.05 | -1.08 | -1.54 |
| 1DAN | T | 44 | D | 0.7 | 0.89 | 0.04 | -0.11 |
| 1DAN | T | 45 | W | 1.6 | 1.05 | -0.09 | -0.25 |
| 1DAN | T | 46 | K | 0.25 | 0.11 | -0.89 | -1.22 |
| 1DAN | T | 47 | S | 0.05 | 0.6 | -0.8 | -1.37 |
| 1DAN | T | 48 | K | 0.4 | 0.43 | -0.42 | -0.46 |
| 1DAN | T | 50 | F | 0.4 | 2.61 | -0.43 | -0.52 |
| 1DAN | T | 51 | Y | -0.1 | 0.58 | -1.27 | -1.58 |
| 1DAN | T | 58 | D | 2.18 | 1.09 | 0.43 | 0.12 |
| 1DAN | T | 61 | D | 0.24 | 0.01 | -0.04 | -0.26 |
| 1DAN | T | 76 | F | 1.2 | 0.62 | -0.98 | -1.39 |
| 1DAN | U | 94 | Y | 1 | 2.7 | 0.91 | 0.85 |
| 1DAN | U | 110 | Q | 1.4 | 1.69 | -0.32 | -0.46 |
| 1DAN | U | 128 | E | 0.1 | -0.11 | -1.33 | -1.64 |
| 1DAN | U | 131 | R | 0 | 0.28 | -0.8 | -1.18 |
| 1DAN | U | 132 | T | 0 | 0 | -1.12 | -1.64 |
| 1DAN | U | 133 | L | 0 | 1.62 | -0.69 | -1 |
| 1DAN | U | 135 | R | 0.55 | 0.94 | -0.64 | -0.76 |
| 1DAN | U | 140 | F | 1.5 | 1.54 | -0.78 | -1.06 |
| 1DAN | U | 163 | S | 0 | 0.42 | -1.29 | -1.61 |
| 1DAN | U | 203 | T | 0.1 | 0.22 | -0.94 | -1.38 |
| 1DAN | U | 207 | V | -0.2 | 1.12 | -0.76 | -1.14 |
| 1DAN | U | 208 | E | 0 | 0.28 | -1.09 | -1.29 |
| 1DX5 | M | 34 | F | 2.6 | 1.01 | -1.21 | -1.66 |
| 1DX5 | M | 36 | K | 1.8 | -0.02 | -0.93 | -1.56 |
| 1DX5 | M | 36A | S | -0.2 | 0.57 | -0.96 | -1.53 |
| 1DX5 | M | 38 | Q | 1.4 | 2.31 | -0.14 | -0.15 |
| 1DX5 | M | 65 | L | 1 | 0.55 | -1 | -1.56 |
| 1DX5 | M | 67 | R | 3.4 | -0.01 | -1.02 | -1.5 |
| 1DX5 | M | 74 | T | 0.8 | 0.2 | -0.81 | -1.32 |
| 1DX5 | M | 75 | R | 0.7 | 0.05 | -0.73 | -1.16 |
| 1DX5 | M | 76 | Y | 3 | 2.03 | 0.73 | 0.61 |
| 1DX5 | M | 77A | R | 1.5 | 2.07 | 0.37 | 0.64 |
| 1DX5 | M | 80 | E | 3.4 | -0.06 | -1.02 | -1.71 |
| 1DX5 | M | 81 | K | 1 | 0.8 | -0.62 | -1.06 |
| 1DX5 | M | 82 | I | 2.6 | 1.07 | -0.96 | -1.39 |
| 1DX5 | M | 84 | M | 0.3 | 0.77 | -1.14 | -1.63 |
| 1DX5 | M | 110 | K | 0 | 0.17 | -1.11 | -1.55 |
| 1FC2 | C | 147 | N | 0.6 | 0.61 | -0.88 | -1.07 |
| 1FC2 | C | 150 | I | 2.2 | 0.78 | -1.11 | -1.53 |
| 1FC2 | C | 154 | K | 1.2 | 0.16 | -1.16 | -1.55 |
| 1FCC | C | 25 | T | 0.24 | 0.1 | -1.01 | -1.37 |
| 1FCC | C | 27 | E | >4.90 | 3.14 | 1.26 | 1.33 |
| 1FCC | C | 28 | K | 1.3 | 0.98 | 1.45 | 1.11 |
| 1FCC | C | 31 | K | 3.5 | 1.91 | 1.27 | 0.74 |
| 1FCC | C | 35 | N | 2.4 | 1.18 | 0.68 | 0.61 |
| 1FCC | C | 40 | D | 0.3 | -0.15 | -0.31 | -0.68 |
| 1FCC | C | 42 | E | 0.4 | 0.03 | -1.07 | -1.34 |
| 1FCC | C | 43 | W | 3.8 | 2.71 | 0.24 | 0.06 |
| 1GC1 | C | 23 | S | 0.29 | 0.27 | -1.2 | -1.87 |
| 1GC1 | C | 25 | Q | 0.03 | 0.42 | -0.94 | -1.29 |
| 1GC1 | C | 27 | H | 0.28 | 0.8 | -0.73 | -1.2 |
| 1GC1 | C | 29 | K | 0.59 | 2.46 | -0.33 | -0.62 |
| 1GC1 | C | 32 | N | 0.18 | 0 | -1.29 | -2.03 |
| 1GC1 | C | 33 | Q | 0.1 | 0.03 | -0.84 | -1.52 |
| 1GC1 | C | 35 | K | 0.32 | 0.55 | -0.07 | -0.31 |
| 1GC1 | C | 40 | Q | -0.41 | 1.66 | 0.23 | -0.06 |
| 1GC1 | C | 42 | S | 0 | 0.03 | 0.62 | 0.2 |
| 1GC1 | C | 44 | L | 1.04 | 0.07 | -0.17 | -0.94 |
| 1GC1 | C | 45 | T | -0.15 | 0.32 | -0.08 | -0.7 |
| 1GC1 | C | 52 | N | 0.7 | 1.01 | -0.84 | -1.34 |
| 1GC1 | C | 59 | R | 1.16 | 1.02 | 0.32 | 0.67 |
| 1GC1 | C | 60 | S | -0.09 | 0.14 | -1.18 | -1.94 |
| 1GC1 | C | 63 | D | -0.32 | -0.05 | -0.95 | -1.51 |
| 1GC1 | C | 64 | Q | 0.44 | 1.02 | -1.24 | -1.83 |
| 1GC1 | C | 85 | E | 1.31 | 2.1 | -1.01 | -1.71 |
| 1JCK | B | 20 | T | 1.4 | 1.26 | -0.3 | -0.64 |
| 1JCK | B | 23 | N | >2.50 | 1.96 | -0.23 | -0.32 |
| 1JCK | B | 26 | Y | 1.7 | 0.92 | -0.45 | -0.82 |
| 1JCK | B | 60 | N | 1.3 | 0.82 | -1.09 | -1.53 |
| 1JCK | B | 90 | Y | >2.50 | 1 | -0.37 | -0.84 |
| 1JCK | B | 91 | V | 2.1 | 1.04 | -0.54 | -0.92 |
| 1JCK | B | 103 | K | 0.4 | -0.26 | -0.79 | -1.24 |
| 1JCK | B | 176 | F | 1.9 | 0.63 | -1 | -1.38 |
| 1JCK | B | 210 | Q | >2.50 | 1.12 | -0.82 | -1.13 |
| 1JTG | A | 104 | E | 1.6 | 2.1 | 1.9 | 2.41 |
| 1JTG | A | 105 | Y | -0.2 | 4.49 | 2.37 | 2.69 |
| 1JTG | A | 130 | S | 0.3 | -0.18 | 0.21 | 0.05 |
| 1JTG | A | 234 | K | 1 | 0.6 | -0.32 | -0.61 |
| 1JTG | A | 235 | S | 1.3 | 2.33 | -0.23 | -0.31 |
| 1JTG | A | 243 | R | 1.3 | 3.76 | -0.22 | 0.11 |
| 1JTG | B | 49 | D | 1.8 | 6.42 | 2.39 | 3.67 |
| 1JTG | B | 74 | K | 3.6 | 0.68 | 0.39 | 0.46 |
| 1JTG | B | 142 | F | 2.1 | 3.23 | 1.26 | 1.27 |
| 1JTG | B | 143 | Y | 0.4 | 0.83 | 0.15 | -0.15 |
