# Supplementary material for: Prediction of hot spot residues at protein-protein interfaces by combining machine learning and energy-based methods
Source: BMC Bioinformatics. 2009 Oct 30;10:365. doi: 10.1186/1471-2105-10-365 (PMC2777894; doi:10.1186/1471-2105-10-365)
Supplement: Additional file 4 — The BID database. Some considerations on the BID database, data set of alanine mutations extracted from it and summary of results obtained with our method. [file 1471-2105-10-365-S4.doc]

**Additional file 4**

**The BID database**

The BID (Binding Interface Database, http://tsailab.org/BID/) is a database of protein-protein interactions that reports information on the effects of mutations. The effect of a mutation is classified as Strong, Intermediate, Weak, Negative, Negative-Strong, Negative-Intermediate, Negative-Weak or Insignificant, on the basis of experiments measuring kinetic and/or thermodynamic quantities. For some entries the change in the free energy of binding (ΔΔG) is available. In general the same entries can be found also in ASEdb (http://nic.ucsf.edu/asedb/), apart from some cases such as mutations in pdb codes 1FCC, 1DX5 and 1FAK. We have included both 1FCC and 1DX5 in our dataset (see table S1) but we missed 1FAK.

In recent publications (e.g. Darnell et al 2007, Cho et al 2009 and Tuncbag et al 2009) mutation data extracted from the BID have been used to test proposed hot spot prediction models (for simplicity we refer to these data sets generally as BID-KFC from now on, from the KFC paper of Darnell et al 2007). Residues for which the effect of an alanine mutation has been classified as ‘Strong’ in BID were considered as hot spots. In our opinion it is questionable whether data such as BID-KFC can be useful to reliably estimate the accuracy of a hot spot prediction method. We detail below are concerns.

The majority of entries in BID-KFC have only kinetic data (e.g. dissociation constant K_d_) associated to them. It is not clear how the BID curators assigned each of these entries to the classification scheme above, whether they have been consistent and how these entries relate to entries classified according to ΔΔG values (e.g. how does a "Strong" assigned on the basis of a kinetic measurement compare to a "strong" from a DDG measurement?). We underline that hot spots are defined on the basis of ΔΔG and this type of measurements should be used to assess a prediction method. It is doubtful whether one can train a model with ΔΔG values and then use it to predict, e.g., K_d_ values.

There are some additional considerations that need to be taken into account

a) BID is no longer maintained which rises a question as to the reliability of the information in it as it is no longer updated and errors are not amended.

b) BID has been substituted by a wikiBID (<http://tsailab.org/wikiBID/index.php/Main_Page>) but it is quite arduous to extract the relevant information. For example, we have not been able to find a number of entries reported in the BID-KFC (Darnell et al 2007). We have found discrepancies in some other cases.

c) BID classifies the effects of a mutation according to a qualitative and subjective scheme (e.g. 'Strong' or 'Weak'). It seems fair to deduce that only a qualitative assessment of a prediction method can be inferred from it.

d) The BID classification does not appear to be fully consistent (at least in the context of hot spot prediction). In cases where the measured ΔΔG is available we have found examples of mutations with ΔΔG > 2 kcal/mol classified as 'Intermediate' and mutations with ΔΔG < 2 classified as 'Strong'.

e) Point (d) raises the further issue of whether it is appropriate to identify hot spot with 'strong' mutations as in KFC or rather hot spots should also include 'Intermediate' mutations.

f) BID-KFC contains several instances of protein-peptide interactions. On the other hand, the vast majority of entries in ASEdb (and all the ones in our data set) relate to protein-protein interactions by means of an extended interface. The two types of interactions are different and involve different mechanisms, e.g. a peptide is clearly much more flexible than a domain. In general it can not be expected that a method designed for one case would work in the other as well. This is particularly true for our method as for example it relies heavily on the environment inter-molecular energy components. The latter term is hardly present in a peptide.

Despite the above considerations, for completeness we have extracted from BID our own data set and tested our method on it. We have included only protein-protein interactions involving an extended interface and excluded protein-peptide interactions. The data set consists of 9 protein complex structures (see Table S7) with a total of 41 mutations. Data set S2 lists the mutations with the associated strength classification as reported in BID. In one case (pdb code 1FAK) the ΔΔG value is also available and we report it. We show also the predictions we obtain using the Transductive SVM (TSVM) model.

Results are summarized in Table S8. When hot spots are identified with strong mutations, results are poor. If instead both strong and intermediate mutations are considered as hot spots, they improve substantially (the F1 score is roughly the same as the one estimated on our original dataset). Given the small size of the dataset it is not clear how significant they are. However it is reassuring that for the one complex for which ΔΔG values are available (1FAK, 19 mutations), predictions are quite accurate (2 true positives, 15 true negative, 2 false positive and 0 false negative).

The complex 1FAK is not homologous to any complex in our training data set.

**Table S7**: Data set of protein complexes and number of mutations extracted from BID.

| PDB  ID | Chain  protein 1 | Chain  Protein 2 | Number of  mutations  Protein 1 | Number of  mutations  Protein 2 |
| --- | --- | --- | --- | --- |
| 1ES7 | AC | BD | 3 | 0 |
| 1FAK | HL | T | 0 | 19 |
| 1FE8 | A | LH | 4 | 0 |
| 1FOE | A | B | 0 | 1 |
| 1GL4 | A | B | 7 | 0 |
| 1MQ8 | A | B | 1 | 0 |
| 1NFI | AB | F | 0 | 2 |
| 1NUN | A | B | 3 | 0 |
| 1UB4 | A | C | 0 | 1 |

**Table S8**: Summary of results for the data set extracted from BID. P=precision, R=recall, F1=F1 score, MCC= Matthews correlation coefficient.

| Hot spot identification | P | R | F1 | MCC |
| --- | --- | --- | --- | --- |
| Str | 0.31 | 0.40 | 0.35 | 0.10 |
| Int or Str | 0.77 | 0.55 | 0.61 | 0.38 |

**Data set S2:** Data set of alanine mutations extracted from BID. We report both the classification of the effects of a mutation (Int=Intermediate, Str=Strong, Insg=Insignificant) and the measured ΔΔG, when available. Our prediction from the TSVM model are also shown (positive scores are residues predicted to be hot spot, i.e. for which ΔΔG ≥ 2 kcal/mol)

| **Pdb id** | **Chain** | **PDB residue number** | **Residue type** | **Effect of mutation** | **Observed ΔΔG (kcal/mol)** | **TSVM score** |
| --- | --- | --- | --- | --- | --- | --- |
| 1ES7 | A | 26 | V | Int | NA | -1.21 |
| 1ES7 | A | 31 | W | Str | NA | -0.71 |
| 1ES7 | A | 49 | F | Int | NA | 0.25 |
| 1FAK | T | 17 | T | Insg | 0.1 | -1.48 |
| 1FAK | T | 18 | N | Insg | 0.2 | -1.46 |
| 1FAK | T | 20 | K | Str | 2.6 | 0.07 |
| 1FAK | T | 22 | I | Weak | 0.7 | -0.80 |
| 1FAK | T | 24 | E | Weak | 0.7 | -1.21 |
| 1FAK | T | 37 | Q | Weak | 0.6 | -0.74 |
| 1FAK | T | 41 | K | Insg | 0.3 | -1.49 |
| 1FAK | T | 42 | S | Insg | 0 | -1.22 |
| 1FAK | T | 44 | D | Weak | 0.8 | 0.06 |
| 1FAK | T | 47 | S | Insg | 0 | -1.32 |
| 1FAK | T | 48 | K | Insg | 0.4 | -0.45 |
| 1FAK | T | 50 | F | Insg | 0.4 | -0.90 |
| 1FAK | T | 58 | D | Str | 2.0 | 0.27 |
| 1FAK | T | 94 | Y | Int | 1.0 | 0.71 |
| 1FAK | T | 133 | L | Insg | 0 | -0.81 |
| 1FAK | T | 135 | R | Insg | 0.5 | -0.79 |
| 1FAK | T | 140 | F | Int | 1.3 | -1.11 |
| 1FAK | T | 203 | T | Insg | 0 | -0.17 |
| 1FAK | T | 207 | V | Insg | -0.2 | -1.67 |
| 1FE8 | A | 963 | R | Insg | NA | 0.24 |
| 1FE8 | A | 987 | E | Insg | NA | -0.14 |
| 1FE8 | A | 990 | H | Insg | NA | 0.47 |
| 1FE8 | A | 1023 | H | Insg | NA | -1.49 |
| 1FO8 | B | 41 | S | Int | NA | 0.31 |
| 1GL4 | A | 403 | R | Int | NA | -0.06 |
| 1GL4 | A | 427 | D | Str | NA | -1.21 |
| 1GL4 | A | 429 | H | Str | NA | 1.43 |
| 1GL4 | A | 431 | Y | Str | NA | 0.32 |
| 1GL4 | A | 440 | Y | Int | NA | 1.05 |
| 1GL4 | A | 616 | E | Str | NA | -1.13 |
| 1GL4 | A | 620 | R | Str | NA | -0.13 |
| 1MQ8 | B | 206 | T | Str | NA | -0.49 |
| 1NFI | F | 181 | Y | Str | NA | -0.56 |
| 1NFI | F | 215 | C | Insg | NA | -0.99 |
| 1NUN | A | 76 | D | Int | NA | 1.93 |
| 1NUN | A | 78 | R | Int | NA | 0.82 |
| 1NUN | A | 155 | R | Int | NA | -0.58 |
| 1UB4 | C | 453 | F | Insg | NA | -1.27 |
